# Rational identification and characterisation of peptide ligands for targeting polysialic acid

**DOI:** 10.1038/s41598-020-64088-z

**Published:** 2020-05-06

**Authors:** Divya G. Shastry, Flaviyan Jerome Irudayanathan, Asher Williams, Mattheos Koffas, Robert J. Linhardt, Shikha Nangia, Pankaj Karande

**Affiliations:** 1 Department of Biological Sciences, Rensselaer Polytechnic Institute, Troy, NY 12180 USA; 2 Center for Biotechnology and Interdisciplinary Studies, Rensselaer Polytechnic Institute, Troy, NY 12180 USA; 3 Department of Biomedical and Chemical Engineering, Syracuse University, Syracuse, NY 13244 USA; 4 Howard P. Isermann Department of Chemical and Biological Engineering, Rensselaer Polytechnic Institute, Troy, NY 12180 USA; 5 Department of Chemistry and Chemical Biology, Rensselaer Polytechnic Institute, Troy, NY 12180 USA; 6 Department of Biomedical Engineering, Rensselaer Polytechnic Institute, Troy, NY 12180 USA

**Keywords:** Biophysics, Biotechnology, Molecular engineering, Lectins, Glycobiology, Peptides, Biochemistry, Carbohydrates, Polysaccharides, High-throughput screening, Biochemical assays

## Abstract

The alpha-2,8-linked form of the polysaccharide polysialic acid (PSA) has widespread implications in physiological and pathological processes, ranging from neurological development to disease progression. Though the high electronegativity and excluded volume of PSA often promotes interference of biomolecular interactions, PSA-binding ligands have important implications for both biological processes and biotechnological applications. As such, the design, identification, and characterisation of novel ligands towards PSA is critical for expanding knowledge of PSA interactions and achieving selective glycan targeting. Here, we report on a rational approach for the identification of alpha-2,8-PSA-binding peptides, involving design from the endogenous ligand Siglec-11 and multi-platform characterisation of peptide binding. Microarray-based examination of peptides revealed charge and sequence characteristics influencing peptide affinity to PSA, and carbohydrate–peptide binding was further quantified with a novel fluorescence anisotropy assay. PSA-binding peptides exhibited specific binding to polymeric SA, as well as different degrees of selective binding in various conditions, including competition with PSA of alternating 2,8/9-linkages and screening with PSA-expressing cells. A computational study of Siglec-11 and Siglec-11-derived peptides offered synergistic insight into ligand binding. These results demonstrate the potential of PSA-binding peptides for selective targeting and highlight the importance of the approaches described herein for the study of carbohydrate interactions.

## Introduction

Polysialic acid (PSA) is a unique polysaccharide playing critical roles in a number of physiological and pathological processes. PSA occurs naturally in a variety of forms, comprising different sialic acid monomers and glycosidic linkages^1,2^. Of the three homopolymeric forms of N-acetylneuraminic acid (the most common sialic acid in humans), only α-2,8-linked PSA is found in our species^1–3^. The roles of PSAs across biology are diverse; humans display highest PSA expression on neural stem and progenitor cells, where expression primarily guides neurological development, but in other systems and organisms, this biopolymer is implicated in bypassing immune surveillance, promoting tissue development, enabling cell migration, and even possibly serving as a molecular reservoir^1–14^. Changes in polysialylation on proteins and cell surfaces are often tied to functional states. For example, change in PSA expression during human embryonic development influences neuronal growth and migration, while in adults, long-chain PSA is largely restricted to regions of the nervous system undergoing remodelling^1–5^. In pathological context, PSA is of great interest due to its occurrence on pathogenic bacteria (*e.g*., *Neisseria meningitidis* groups B and C), where expression in the polysaccharide capsule enables evasion of the host immune system^1,2,9–12^. Additionally, PSA has been found on tumour cells, and its expression has been correlated with poorer prognosis of certain cancers, possibly due to an increase in metastatic potential^1,2,13^. Despite the broad diagnostic and therapeutic application space available with the ability to understand, target, and detect PSA, *in vitro* or *in vivo*, few studies have sought to develop and characterise robust ligands against PSA.

Several naturally occurring proteins have been identified to bind to PSA, including neurotrophins and polysialyltransferases^1,2^. However, information (*e.g*., residue-level and structural) about direct interaction of PSA with endogenous ligands is limited^14–23^. Additionally, challenges in protein isolation and stability deter characterisation and applicability of such ligands for PSA targeting. The generation of anti-PSA antibodies, together with their biochemical characterisation (as of mAb735), has proved useful^2,21,22^. Yet, as with native ligands, factors including production, size, and stability may limit the versatility of antibodies in applications; these factors comparatively promote the versatility of low molecular weight ligands. Besides such advantages for the latter ligands (described elsewhere^24^), the identification of small targeting agents, like peptides, for PSA can add to basic knowledge of PSA molecular interactions, especially when rational design strategies are incorporated in ligand development. Furthermore, ligand design with a focus on elucidating binding characteristics can provide critical information for iterative design and future targeting efforts.

In this study, we report on the design and identification of α-2,8-PSA-binding peptides, with biochemical and thermodynamic characterisation of peptide binding and insight into PSA interaction with the endogenous ligand Siglec-11^23^. We have previously identified peptide ligands binding to α-2,8-PSA, demonstrating the success of a high-throughput peptide microarray platform for evaluating design strategies and supporting preliminary peptide characterisation^25^. Here, we examined amino acid sequences derived from a naturally occurring lectin to PSA in tandem with previously identified peptides, while simultaneously expanding on peptide characterisation using a synergistic experimental and computational approach.

## Results

### Library-level examination of peptide–PSA interaction

Peptides (762 unique sequences) were screened for binding to α-2,8-PSA using a high-throughput microarray screening platform^25^. A library of peptides was designed from linear epitope mapping of the protein Siglec-11 (all domains) with peptides 15 residues in length overlapping by 13 residues (329 peptides; 1 previously reported^25^). Siglec-11 is a member of the highly homologous Siglec family of sialic acid binding-proteins with 2,8-linkage specificity^23,26–28^. An additional 172 peptides (1 previously reported^25^) were designed based on Siglec-derived sequences. Modifications to these peptides included select rational mutations and sequence scrambling. Siglec-modified sequences designed with preliminary binding hypotheses were synthesized alongside the parent library; other sequences based on screening of the original library were synthesized iteratively, where binding and non-binding peptides were modified to study variation in target affinity and selectivity. Random sequences from *de novo* design and from prior reports in literature^29^ (38 peptides), as well as PSA-binding and non-binding peptides previously designed from mAb735 and phage display screening (223 peptides)^25^, were concurrently screened for intra-assay comparison to binding of Siglec-derived peptides.

**Figure 1:**
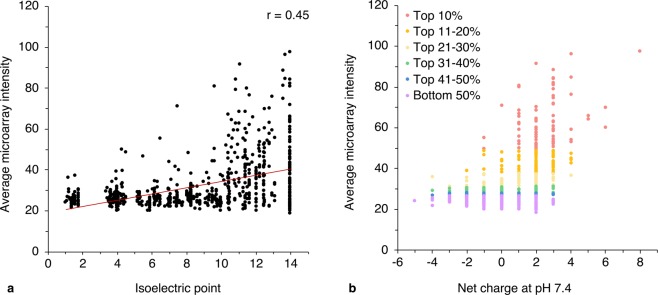
Binding of peptides to α-2,8-PSA based on **(a)** isoelectric point (pI) or **(b)** net charge at pH 7.4. **(a)** Peptide binding intensity with pI displays a very weak correlation (r = 0.45) due to a large number of high pI peptides demonstrating lack of interaction with PSA. All peptides plotted with a pI of 14 displayed the output “>14” with EMBOSS iep. **(b)** Charge-based binding for 10% intensity divisions of the peptide library are shown. The outlying point with net charge = 8 represents a *de novo* designed peptide with alternating Lys and Gly residues. Binding intensities represent the mean of three independent experiments, with triplicate intra-assay measurements (error bars excluded for clarity).

Sequences of peptides exhibiting the highest (approximately top 5%) binding intensities from the complete peptide library are provided in Table 1. As expected, all sequences display a prevalence of positively charged residues. The charge dependence on binding at the library-level is apparent from Fig. 1, which displays an increase in microarray binding with higher peptide basicity and charge. However, a few neutral and negatively charged peptides display measurable affinity towards PSA, and not all positively charged peptides interact with PSA; this suggests that observed binding cannot be attributed to non-specific electrostatic interactions alone. Differentiating peptides based on binding, charge, and origin does not indicate that of the various peptide development strategies chosen, one provides a distinct advantage in increasing charge-based peptide affinity (Supplementary Fig. S1).

**Table 1:** Sequences and origins of 25 high-binding peptides from microarray screening against α-2,8-polysialic acid. Peptides shown exhibit binding intensities in the top 5% in three independent screens, with triplicate measurements within each screen and inter-assay coefficients of variation <25% (peptides exhibiting intensities in the top 5% with higher inter-assay CVs excluded). Bolded residues represent mutations from parent peptides. * Peptides with selectivity >80%.

Peptide index	Peptide sequence	Peptide origin
I-P23	YWFKGRTSPKTGAPV	Siglec-11
I-P24*	FKGRTSPKTGAPVAT	Siglec-11
I-P59	LSNAFFLKVTALTKK	Siglec-11
I-P60	NAFFLKVTALTKKPD	Siglec-11
I-P62	LKVTALTKKPDVYIP	Siglec-11
I-P85	AALSPRRTRPSTSHF	Siglec-11
I-P86	LSPRRTRPSTSHFSV	Siglec-11
I-P102	VDFSRKGVSAQRTVR	Siglec-11
I-P103	FSRKGVSAQRTVRLR	Siglec-11
I-P104	RKGVSAQRTVRLRVA	Siglec-11
I-P143	WGPRTLGLELRGVRA	Siglec-11
I-P278	FRVKICRKEARKRAA	Siglec-11
I-P279	VKICRKEARKRAAAE	Siglec-11
I-P330	WFKGRTSPKTGAPVA	Siglec-11
I-P333	WFKG**K**TSPKTGAPVA	Siglec-11: R5K mutation
I-P378	KGKGKGKGKGKGKGK	*De novo*
I-P379	KGGGKGGGKGGGKGG	*De novo*
I-P380	GGKGGGKGGGKGGGK	*De novo*
II-P35	GSGSGTDFTLKISRV	mAb735
II-P50	VPYTFGGGTRLEIKG	mAb735
II-P77	PGSGNTKYNEKFKGK	mAb735
II-P79	NTKYNEKFKGKATLT	mAb735
II-P213	AISSPLL**R**NPFRGGGS	Phage display screening: W8R mutation
II-P214*	AISSPLL**K**NPFRGGGS	Phage display screening: W8K mutation
II-P336*	NRTVL**R**NLGNGTSLP	Siglec-11: E6R mutation

**Figure 2:**
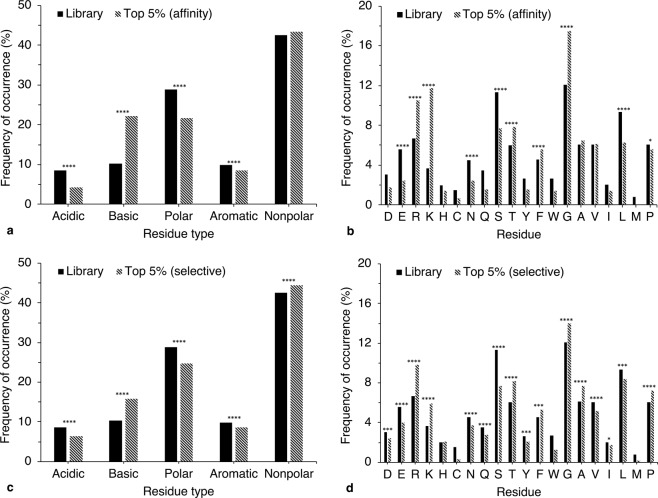
Compositional analysis of high affinity **(a,b)** and high selectivity **(c,d)** peptides. Occurrence of residue types **(a,c)** or specific residues **(b,d)** in the peptide library (n = 762) is compared to occurrence in approximately the top 5% affinity or selectivity peptides (n = 38 or 41, respectively). Acidic = D and E; basic = R and K; polar = H, C, N, Q, and S; aromatic = Y, F, and W; and nonpolar = G, A, V, I, L, M, and P. (Two-tailed *z* test for population proportions; *p < 0.05, **p < 0.01, ***p < 0.001, ****p < 0.0001, two-tailed; *p* values *p*rovided in Supplementary Table 1). Statistical significance of frequency changes of the following residues was not determined as low residue occurrence in the sample population precluded the assumption of normal distribution: D, H, C, Q, Y, W, I, and M **(b)** and C, W, and M **(d)**.

The relationship between basic residues and PSA binding is supported by compositional and positional analyses of sequences of high affinity peptides. Figure 2 and Supplementary Fig. S2 display statistically significant increases in basic residues in the top 5% of binders. Several residues show agreement with our prior work on mAb and phage display-derived peptides (specifically, significant increases in the prevalence of arginine, lysine, and phenylalanine and decrease in that of serine)^25^. However, changes in the occurrence of asparagine and glycine were reversed; here, asparagine showed significant decrease and glycine showed significant increase. These differences are likely due to examination of a larger peptide library in this study, as well as inclusion of a larger number of non-phage peptides (lacking the inherent biases in residue propensity observed in phage-derived lead candidates^30^) and restriction of analyses to the top 5% of binders (as compared to the top 10% reported previously).

### Fluorescence anisotropy assay for assessment of peptide binding affinity

A fluorescence anisotropy (FA) assay was developed for the determination of peptide–PSA binding affinity. In FA assays, in-solution binding of an analyte to a smaller, fluorescently-labelled ligand is quantified through titrations with increasing concentrations of the analyte^31^. FA assays have been used to study various interactions of proteins, DNA, carbohydrates, and small molecules^31–34^. However, use of these assays to study peptide–carbohydrate interactions has been limited. In the experiments described here, anisotropy should be interchangeable with polarization; anisotropy is employed as it is normalized by total intensity^31^.

**Figure 3:**
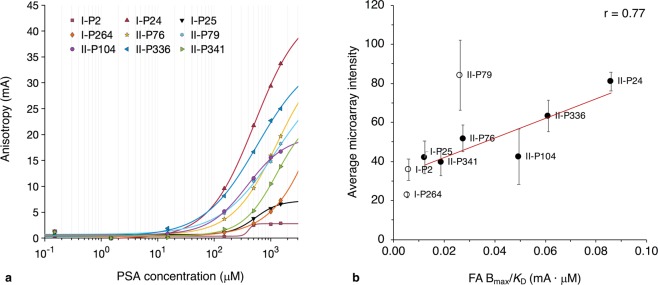
(**a**) Interaction of PSA-binding and non-binding peptides with PSA as assessed by fluorescence anisotropy titrations (solubility limit of PSA ~1.5 mM). Anisotropy values were transformed by subtraction of minimum FA values for each curve (approximately equivalent to anisotropies of free peptides) to enable comparative analysis between peptides. Error bars are excluded for clarity; experimental errors (standard deviations) are provided in Supplementary Table S2 along with non-adjusted anisotropy values. **(b)** Relationship of microarray “affinity” (intensity values) to peptide B_max_/*K*_D_ (from FA assay). Open circles represent peptides excluded from determination of Pearson correlation coefficient due to higher imprecision in B_max_ and *K*_D_ from anisotropy curve fits for these peptides (criteria for exclusion: fit standard error of B_max_ or *K*_D_ > fit value); values for these peptides are represented for comparison of microarray intensities to other peptides. Microarray intensities represent the mean of three independent experiments, with triplicate measurements within each screen. FA *K*_D_’s are from at least two independent experiments, with at least triplicate measurements within each assay. Error from the assay with greater inter-assay variability (*i.e*., microarray screening) is shown (inter-assay standard deviation).

Nine peptides of different origins were selected for study with the FA assay, and binding affinities of fluorescently labelled-peptides to PSA were determined (Fig. 3(a) and Table 2). Peptides were selected, in part, to represent a range of PSA-binding abilities in microarray studies. Binding analysis of I-P50, a peptide from the putative binding region of Siglec-11, could not be performed due to high insolubility of the peptide in aqueous media (peptides modified from I-P50 to increase hydrophilicity also displayed poor solubility). Under the conditions used, peptides bound PSA in approximately 1:1 ratio. Given this stoichiometry, together with the high conformational flexibility of peptides and PSA^35^ (as compared to proteins or monosaccharides), the mid-to-high micromolar affinity values obtained are not unexpected.

**Table 2:** Binding affinity constants (*K*_D_’s) of peptides as determined by FA assays. SE = standard error of the fit (for *K*_D_) with 95% confidence bounds. R square values of curve fits are also provided. Bolded residues represent mutations from parent peptides. Accuracy of values marked * is likely poor due to high imprecision (SE > *K*_D_ for II-P79 and SE >> *K*_D_ for I-P2 and I-P264); corresponding peptides are excluded from correlation in Fig. 6(b).

Peptide index	Peptide sequence	Peptide origin	*K*_D_ (mM)	SE (mM)	R^2^
I-P2	KDPSYSLQVQRQVPV	Siglec-11	0.405*	13.896	0.955
I-P24	FKGRTSPKTGAPVAT	Siglec-11	0.521	0.077	1.00
I-P25	GRTSPKTGAPVATNN	Siglec-11	0.536	0.174	0.977
I-P264	EHGGGLGLGAALGAG	Siglec-11	9.85*	53.43	0.994
II-P76	IYPGSGNTKYNEKFK	mAb735	1.29	0.70	0.998
II-P79	NTKYNEKFKGKATLT	mAb735	1.20*	1.31	0.995
II-P104	HLSLKNPLRMDLGGGS	Phage display screening	0.388	0.091	0.997
II-P336	NRTVL**R**NLGNGTSLP	Siglec-11: E6R mutation	0.577	0.164	0.999
II-P341	NRTVLE**K**LGNGTSLP	Siglec-11: N7K mutation	1.29	0.886	0.995

*K*_D_ determinations confirmed microarray-based classification of peptides as PSA-binding or non-binding. Peptide affinity from microarray studies represents intensity values and is not equivalent to affinity constants; hence, it is expected that microarray affinity does not correspond exactly with *K*_D_ values. Microarray intensities show strong correlation to anisotropy values (and not *K*_D_ itself) at higher PSA concentrations, with r ≈ 0.8 at concentrations ⪆100 μM (at lower concentrations, higher relative error in anisotropy prevents accurate assessment of correlation). I-P264 was used as a negative control^25^ and displays the weakest binding, with *K*_D_ > 1.5 mM (binding does not approach saturation even at the solubility limit of PSA). Along with microarray screening, SPR spectroscopy^25^ and molecular dynamics (MD) simulations (see below) corroborated the non-binding property of this peptide.

The ratio B_max_/*K*_D_ shows strong correlation to microarray intensities (r = 0.77; Fig. 3(b)). Though *n* is low, this relationship demonstrates that the identification of PSA-binding peptides through selection of high intensity binders on microarrays is likely to isolate peptides of moderate-to-high “binding potential,” *i.e*., peptides with high B_max_ and/or low *K*_D_. Hence, quantification of binding potential effectively enables selection of a peptide candidate pool which captures two potentially desired properties in peptide ligand applications. (The term binding potential is derived from PET imaging; use of the term here does not indicate any relationship to this technique^36^). Of the peptides characterised with FA, I-P24 and II-P336 notably display the highest B_max_/*K*_D_ ratios and reproducibly high microarray affinity intensities (Fig. 3(b)). Since B_max_/*K*_D_ is roughly equivalent to the initial slope of the binding curve, a higher ratio generally corresponds to a greater response with a smaller amount of target, which may be useful in assays where peptide ligand sensitivity is critical.

### Assessment of peptide selective binding to α-2,8-PSA

Competitive microarray screening with α-2,8/9-PSA was conducted in order to assess selective binding of peptides to α-2,8-PSA. The definition of percent selectivity at 10% competing glycan was applied to enable selectivity comparisons amongst a larger set of peptides^25^ (nearly all peptides displayed minimal binding to α-2,8-PSA with equimolar α-2,8/9-PSA). In contrast to peptide affinity analysis, where higher positive charge and affinity generally correlated, selectivity analysis of high affinity peptides indicated that affinity is not sufficient to enable selective binding. We have previously demonstrated this for selective binding of peptides to PSA over chondroitin sulphate^25^. However, the use of an isomeric polymer is a more stringent assessment of selectivity; here, selective binding is guided by three-dimensional conformation and not additionally by differences in functional groups.

Of the high affinity peptides in Table 1, peptides with selectivity greater than 80% are I-P24, II-P214, and II-P336. (Selectivities of high affinity peptides and of peptides with selectivities >80% are provided in Supplementary Table S3.) These three peptides have different origins. I-P24 is derived from the N-terminal binding domain of Siglec-11. II-P214 was designed with a lysine point mutation to modify binding of a previously discovered non-binding peptide from phage display screening. Similarly, II-P336 was designed from a non-binding Siglec-derived peptide through substitution of glutamic acid with arginine. In the second two cases (where poor binders were modified), distinctly separated positive charges were incorporated in peptide regions lacking positive charge; charge spacing was based on preliminary hypotheses from principles of lectin–carbohydrate binding^37–42^. Of these three peptides, only I-P24 consistently displayed high affinity binding in the top 2% of microarray intensities. Other than I-P24 and II-P336, peptides characterised with FA showed moderate to poor binding to α-2,8-PSA in the presence of different concentrations of α-2,8/9-PSA (Supplementary Fig. S2). Interestingly, I-P24 and II-P79 (from mAb735 and also assessed with FA) demonstrated markedly differing affinity and selectivity responses as compared to their respective overlapping peptide sequences (Supplementary Fig. S2), suggesting sequence-specific PSA binding by these peptides.

Peptides of higher selectivity have highly similar residue composition to peptides of higher affinity, except for a relatively lower propensity of lysine in selective sequences (Fig. 2). This similarity is expected as some degree of affinity is necessary for selective binding to occur, and hence, selective binding was assessed in peptides displaying binding above background level. However, positional occurrences of residues differ in high affinity and high selectivity sequences. In selective sequences, some positions display a reduction in positively charged residues, while others show an increase in small, hydrophobic residues (Supplementary Fig. S3 and Supplementary Table S4). This aligns with the observation that the highest affinity peptides do not necessarily bind selectively to PSA; such sequences contain numerous positively charged residues that may bind indiscriminately to any negatively charged target. Thus, increased net charge promotes peptide affinity to PSA, but may not enhance selective binding. In fact, *de novo* peptides composed of lysine and glycine residues display high affinities but mediocre selectivity (approximately 55–60%, *e.g*., I-P378–I-P380 in Table 1). With the additional consideration that selectivity quantification was performed with only 10% competing PSA, these highly basic peptides provide poor examples of applicable selectivity.

**Figure 4:**
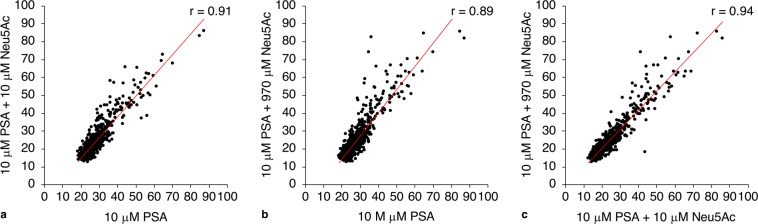
Selective binding of peptides to polymeric sialic acid over sialic acid monomer. Mean microarray intensities are compared between peptide binding to **(a)** 10 μM PSA and 10 μM PSA with equimolar (10 μM) Neu5Ac, **(b)** 10 μM PSA and 10 μM PSA with equivalent mass (970 μM) of Neu5Ac, and **(c)** 10 μM PSA with equimolar Neu5Ac and 10 μM with equivalent mass Neu5Ac. Given that PSA contains an average of 100 sialic acid units, competition of a mass equivalent of Neu5Ac represents a more stringent condition than equimolar competition. Binding intensities represent the mean of two independent experiments, with triplicate intra-assay measurements (error bars excluded for clarity).

In contrast to the narrow definition of selectivity above, specificity of peptides to polymeric α-2,8-PSA was demonstrated through competitive microarray screening with N-acetylneuraminic acid (Neu5Ac or sialic acid monomer). Peptide library binding to PSA in the presence of either molar or mass equivalents of Neu5Ac showed strong or very strong correlations (Fig. 4). As proof-of-principle, the FA assay developed for peptide–PSA affinity assessment was applied for I-P24 and I-P264 with Neu5Ac, and the specific interaction of I-P24 with the polymer was confirmed (Supplementary Fig. S4).

Variation in binding between Neu5Ac conditions largely fell within assay error. However, a few peptides demonstrated higher binding to PSA with a mass equivalent of Neu5Ac, possibly due to effects of solution charge and ionic strength on binding. The microarray technique likely lacks the sensitivity needed for systematic evaluation of such effects (*i.e*., compared to techniques such as FA or isothermal calorimetry [ITC]^43^); however, preliminary assessment of selective binding in differing buffer conditions is possible. Affinity screening of a sub-set of peptides with 100 mM NaCl (the standard buffer concentration) and with 200 mM NaCl did not reveal a clear trend for altered binding with higher ionic strength (r = 0.84). I-P2, I-P24, I-P25, and I-P264 (tested with FA) and I-P50 (discussed further below) were included in this sub-set, and these peptides did not display any change in binding (within error). In contrast, nearly all peptides showed minimal binding to PSA when the affinity screen was conducted without NaCl; I-P24 was one of the few peptides displaying detectable binding. It is possible that this large decrease in binding arises from elimination of a positive entropic contribution, similar to the energetic contribution previously described for binding of heparin to peptides, where sodium ion displacement entropically drives the carbohydrate–peptide interaction^37,44^.

Peptide selective binding to PSA at different pH values was also evaluated through microarray screening. Most peptides, including all FA-tested peptides, were largely insensitive to changes in pH in the range 6.0–8.0 (Supplementary Fig. S5). Peptides exhibiting the most prominent differences between conditions contain histidine residues, for which change in protonation at lower pH expectedly enhances PSA interaction (Supplementary Fig. S5).

### Molecular dynamics simulations of PSA–peptide and PSA–Siglec-11 interactions

**Figure 5:**
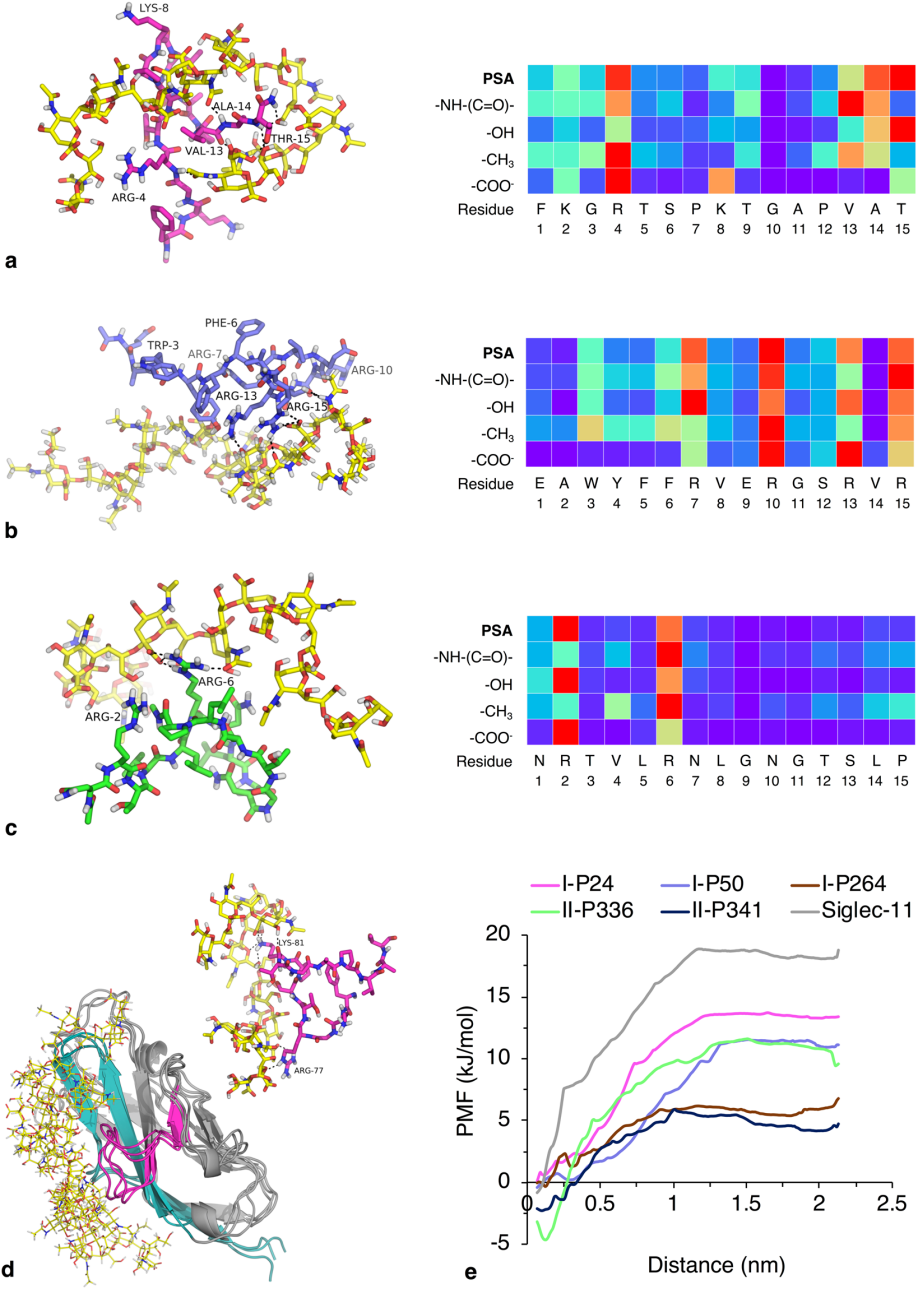
Interactions of peptides and Siglec-11 with α-2,8-PSA as assessed by MD simulations. Simulation snapshots from the time point of greatest interaction (with a cut-off distance for interaction set at 3.0 Å) for **(a)** I-P24, **(b)** I-P50, and **(c)** II-P336 with α-2,8-PSA (decasialic acid) (1:1) are shown on the left. Polar contacts at the time point of the snapshots are shown as black dashes, and residues for which highest interaction occurs over the course of the complete simulation trajectory are labelled. The carbohydrate is shown in yellow, and peptides I-P24, I-P50, and II-P336 are shown in magenta, blue, and green, respectively. Corresponding residue interaction maps (right) show the normalized number of contacts (3.0 Å cut-off distance) from 0 (purple) to 1 (red) observed over the complete time course of simulation between each peptide and decasialic acid. II-P341, a moderate to weak binder in microarray and FA assays, did not show PSA binding in 1:1 simulation, but demonstrated binding in other simulations (see Supplementary Information). **(d)** Siglec-11 N-terminal domain (homology model) with α-2,8-PSA (decasialic acid) (1:1; overlay of three frames with highest interaction). The carbohydrate is shown in yellow in rod model, and the protein is shown in grey in ribbon model with the CC′ loop in magenta and F/G strands in teal. *Upper right*, Snapshot of the CC′ loop alone in rod model in contact with PSA, with polar contacts shown as black dashes. General loop structure and contact residues are highly similar to those of I-P24. Simulation snapshots for **(a–d)** are provided as Supplementary PDB files. **(e)** Potential of mean force (PMF) curves of the five peptides and Siglec-11 binding to α-2,8-PSA (distance along reaction coordinate).

MD simulations of PSA–peptide and PSA–Siglec-11 interactions were performed to investigate thermodynamic and structural aspects of peptide–PSA binding, as well as the relationship of this binding to protein–PSA binding (Fig. 5). Simulations of the five peptides I-P24, I-P50, I-P264, II-P336, and II-P341 (all derived from Siglec-11; Table 2) with α-2,8-PSA largely corroborated microarray and FA observations on PSA–peptide binding, the latter for all but I-P50 (for which poor solubility hindered experimental binding studies). For PSA-binding peptides, interactions were primarily guided by positively charged residues, which align with sialic acid carboxyl groups. Additionally, proximal residues appeared capable of forming hydrogen bonds and Van der Waals contacts with PSA. I-P264, the negative control peptide, showed minimal interaction with PSA.

Simulations of the N-terminal binding domain of Siglec-11 (homology model) with PSA demonstrated target interaction with CC′ and GG′ loops of the immunoglobulin domain, as well as with the F, G, and G′ strands; the arginine conserved amongst Siglecs projects into the binding pocket from the hydrophobic F strand (part of the domain core) to interact with sialic acid carboxyl groups. I-P24 and I-P50 comprise the CC′ loop and F strand/FG loop, respectively. Attempts at experimental quantification of PSA–Siglec-11 binding through SPR spectroscopy and ELISAs failed due to low affinity; previous studies have successfully identified but not quantified binding^23,26^. Also, high protein concentrations required for low affinity quantification limited analysis through other techniques, such as FA, that were optimized for the study of peptide binding. However, potential of mean force (PMF) curves of Siglec-11 Domain 1 with PSA suggest a micromolar binding affinity, based on comparison to PMF curves of peptides (Fig. 5(c)) and experimental peptide characterisation. Results from MD simulations of the five peptides and Siglec-11 with α-2,8/9-PSA are provided in Supplementary Information (Supplementary Fig. S6).

### Targeting of human neural progenitor cells with Siglec-11-derived peptides

**Figure 6:**
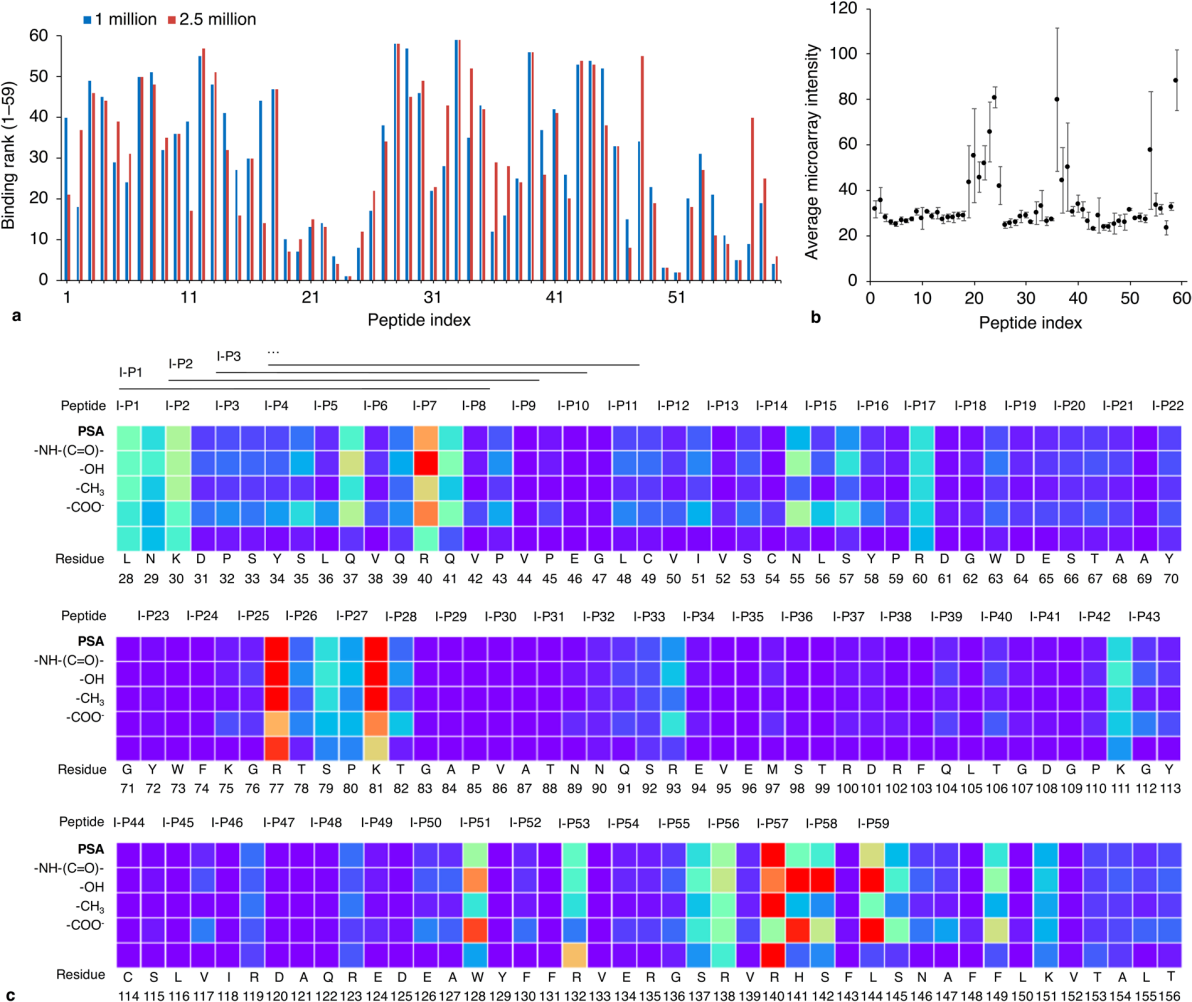
(**a**) Binding to peptides derived from Domain 1 of Siglec-11 (I-P1–I-P59, including residues from linker regions) to NPCs. Binding rank represents ranking of binding intensities of peptides (with a rank of 1 corresponding to the peptide showing highest binding amongst 59 peptides). Intensity data (means) are derived from triplicate measurements for each peptide in screening against 1 or 2.5 million NPCs per microarray (error bars excluded for clarity). ρ = 0.83; p = 0.257, α = 0.05, two-tailed. Screening with 5 million cells could not be accurately quantified due to high microarray background. Peptides derived from the Siglec-11 CC′ loop (residues 74–88) and FG/G region (residues 128–152) demonstrate higher relative binding to NPCs as compared to other peptides, with low rank values for NPC binding (Siglec-11 residue numbering is provided in **(c)**). While a few other peptides display low binding ranks, the specified regions display low ranks for contiguous peptides. **(b)** Microarray binding of I-P1–I-P59 to PSA. Binding intensities represent the mean of three independent experiments, with triplicate intra-assay measurements (error bars show inter-assay standard deviations). **(c)** Interaction map showing the normalized number of contacts (3.0 Å cut-off distance) from 0 (purple) to 1 (red) observed in the MD simulation between Siglec-11 Domain 1 and decasialic acid. Residues correspond to Siglec-11 numbering, and peptide identifiers mark the start of each 15-residue peptide sequence. A similar interaction map for octasialic acid is provided in Fig. S7.

The relative binding of Siglec-11-derived peptides to a PSA-expressing cell line was determined through microarray screening to assess cell-targeting capabilities of PSA-binding peptides. The sub-set of peptides I-P1–I-P59 (from Siglec-11 Domain 1 linear mapping) was selected for cell binding analysis to supplement and elucidate experimental and computational binding data for I-P24. Relative binding of these peptides to human neural progenitor cells (NPCs) is shown in Fig. 6, along with peptide microarray binding and a corresponding residue interaction map or “epitope map” from simulations. There is a significant association between binding ranks for two cell numbers (Spearman correlation coefficient (ρ) = 0.83; p = 0.257, α = 0.05, two-tailed), indicating selective binding of cells to PSA-binding peptides. Importantly, peptides derived from or near i) the CC′ loop and ii) the FG loop and G/G′ strand have prominently lower rank values in comparison to other peptides. The corresponding regions of Siglec-11 contact the PSA ligand in simulations, and some peptides from the FG/G region demonstrated moderate-to-high microarray affinity and moderate selectivity in microarray 2,8/9-PSA competition studies.

## Discussion

The identification of PSA-binding peptides may be pursued using a number of design strategies, random or rational, and residue-level information related to PSA-interactions may be gained through any strategy. However, design from and comparison to the PSA-binding protein Siglec-11 enabled a complementary study of peptide and protein structure and function, wherein i) peptide design and characterisation provided biochemical understanding on PSA-binding, ii) peptide study led to molecular insight into Siglec-11 interaction, and iii) protein and peptide simulations validated experimental peptide characterisation. Simultaneously, the concurrent analysis of peptides of different origin enabled insight into comparative binding characteristics of Siglec-11-derived peptides.

Assessment of peptide affinity with microarray screening enabled characterisation of residue-based binding properties alongside identification of PSA-binding and non-binding peptides. In comparison, subsequent FA titrations enhanced characterisation through direct quantification of peptide–PSA interaction; *K*_D_ and B_max_ determinations allowed for property-based discrimination of peptides, and assessment of binding potential supported peptide selection strategy from microarrays. Such subsequent characterisation validates conclusions from high-throughput screening and, furthermore, promotes informed selection of peptide candidates for desired applications.

Despite limited literature on the use of FA assays for the study of carbohydrate interactions^34^, these assays are an attractive platform for in-solution binding analysis of carbohydrate–peptide interactions, which generally demonstrate low 1:1 affinity^24,37–40,43,45–47^. In general, there are few reports on thermodynamic and/or kinetic characterisation of polysaccharide–peptide binding, especially for interactions with high micromolar affinities^34,48–50^. In many binding analysis techniques, low affinities complicate or prevent accurate *K*_D_ determination, as micromolar and millimolar interactions pose detection challenges^43,46^. Additionally, in some techniques, such as SPR spectroscopy, the viscosity of carbohydrate solutions may result in artifacts. Though PSA–peptide binding was detected in prior work with SPR^25^, accurate affinity quantification with this technique was limited by a combination of factors, including the high viscosity of PSA, the affinity detection limit of the system, and the small size of peptides relative to PSA. In contrast, despite limitations with PSA solubility (upper limit of approximately 1.5 mM) and relatively low anisotropy changes, PSA–peptide binding analysis with FA successfully identified and characterised two PSA-binding peptides (I-P24 and II-P336) which performed well in all other experimental and computational assessments. The entropic penalty of binding associated with a low molecular weight peptide interacting with a conformationally flexible, high molecular weight polysaccharide is likely high; hence, the design and characterisation of PSA-binding peptides with *K*_D_’s comparable to those of Siglec–ligand interactions^51^ is notable.

Select peptides, including I-P24 and II-P336, were shown to display some degree of selective binding to PSA of 2,8-linkages. However, specificity to PSA of these linkages was absent, with binding outcompeted by α-2,8/9-PSA to various degrees. The difference in peptide binding between these isomers, suggested by the selectivity–specificity contrast and supported by MD simulations, may be manipulated to alter selectivity. For example, numerous studies have demonstrated the importance of ligand density in affinity of carbohydrate interactions^37–40,43,52–55^, and there is evidence for altered selectivity with alteration in carbohydrate density^56^; a similar approach could be undertaken wherein ligand density of peptide candidates displaying promising selectivity (with 10% competitor) is modulated to influence selectivity. Furthermore, though peptides with selectivity for α-2,8-PSA were of focus here, peptides binding to α-2,8/9-PSA—absent in humans—may be refined for targeting of pathogenic bacterial species expressing this PSA^1,2^. Similarly, given the ubiquity of sialic acid in mammalian systems^57^, the demonstrated specificity of PSA-binding peptides to polymeric sialic acid over the monomer can provide considerable advantages in *in vitro* or *in vivo* applications.

The pH insensitivity in binding of high affinity and high selectivity PSA-binding peptides, such as I-P24, may be considered another example of selective binding. This selectivity allows for peptide utilization in environments with variable pH values (*e.g*., physiological systems). In contrast, pH sensitive peptides demonstrate that selectivity of peptides to PSA may be engineered to switch, which may be useful in “bind and elute” contexts. For example, PSA-binding peptides may be used to target or purify therapeutic proteins with sialic acid modifications of different degrees of polymerization (DPs).

The differing molecular bases^25,58,59^ of the thus demonstrated PSA–peptide affinity and selectivity was suggested by experimental techniques (employed for peptide identification and characterisation) and supported by computational approaches (used to validate and further characterise binding). For example, compositional and positional analyses of peptides suggested that PSA selectivity requires binding of positively charged residues (preferably Arg, as in protein–carbohydrate interactions^41,42^) appropriately spaced by small, hydrophobic residues; simulations likewise demonstrated that electrostatic interactions guide peptide–PSA binding. The apparent lack of a strict consensus motif may be due to conformational flexibility of both the peptide ligand and carbohydrate target. It is possible that DP of PSA affects peptide/protein binding through, for example, conformational differences, entropic effects, stability of species, or multivalency^14,22,35,60,61^. While experimental studies used a polydisperse polymer (with an average DP of 100) and MD simulations assessed binding with decasialic acid, results from the two methodologies showed overall good agreement—especially for I-P24.

Of the peptides evaluated with simulations, I-P24 and I-P50 originated from the N-terminal binding domain (Domain 1) of Siglec-11. The other peptides were derived directly or modified from non-binding regions of the protein, and one of these peptides (II-P336) displayed good affinity and selectivity to PSA. The experimental performance of Domain 1 peptides, specifically high affinity and selectivity of I-P24 and weak binding of I-P50, can be explained by their origins. I-P24 forms the CC′ loop in Siglec-11^27^. This loop, which is highly variable amongst Siglecs and bordered by conserved beta strands, has been shown to contain specificity determinants in other Siglecs^27,62^. In Siglec-11, the orientation of the CC′ loop is such that it points inwards into the ligand binding pocket as for Siglec-2, -4, -5, and -8^62^. Interestingly, simulations demonstrate that I-P24 also adopts a highly similar omega loop structure on binding PSA. Additionally, the residues of the Siglec-11 loop interacting with PSA are the same residues of I-P24 that orient towards the ligand, with positively charged residues and polar groups contacting the ligand and hydrophobic side chains largely stabilizing the loop’s interior. A central Pro (as with Siglec-4, -5, and -8) results in turning of the loop. In both the Siglec-11 CC′ loop and I-P24, it is possible that this structural feature, combined with smaller residues maintaining loop flexibility, enable proper orientation of Arg and Lys residues for specific ligand binding. Combined with microarray and FA data on I-P24 selective binding to α-2,8-PSA, the Siglec-11 CC′ loop simulations agree with the prevailing view that this loop confers ligand specificity to Siglecs.

Peptides from the Siglec-11 region containing the Arg conserved in Siglecs^27^ (I-P50 and similar) did not experimentally demonstrate high binding, with binding only evident with increased exposure times in microarray imaging. It is possible that microarray binding is partially affected by high peptide hydrophobicity; due to peptide presentation from a partly hydrophobic surface into aqueous media, hydrophilic peptides may be more accessible for target binding. In fact, a few peptides modified from this region to have higher hydrophilicity demonstrated binding in the top 5% of intensities in affinity screening, but also displayed high inter-assay variability. Furthermore, simulations of I-P50 binding to PSA indicate that the interaction, though weak, occurs through guiding electrostatic interactions of Arg residues (corresponding to the conserved Arg from strand F and other Arg residues from the FG loop/G strand in Siglec-11). It is possible that the hydrophobicity of contiguous residues promotes Arg residue binding to PSA when displayed from deeper within an otherwise hydrophilic protein binding site (though, of note, the conserved Arg is thought to be less critical for Siglec-11 binding than for other Siglecs^23^). However, in isolation (*i.e*., in peptide form), the residues composing I-P50 likely lack topological context and hence target specificity. This contrasts directly with the structure of the CC′ loop, which supported the engineering and comparative study of I-P24.

Unlike in microarray affinity screens with PSA, cell screening exclusively identified binding site-derived peptides. Linearly mapped peptides demonstrating affinity to PSA alone did not show consistently greater binding to NPCs, unlike CC′ loop and F/G strand-derived peptides, which is expected as linear epitope mapping is not a surrogate for binding site determination. Thus, as compared to binding and competition assays with few species, cell screening served as a peptide selectivity challenge of greater stringency. Both Siglec-11 simulations and cell binding data suggest that along with the CC′ loop, the F/G strand may play a role in ligand specificity. However, the higher performance of I-P24 over F/G strand-derived peptides in various affinity and selectivity assessments makes this peptide exemplary for characterisation and application. The remarkable difference in binding of I-P24 and other select peptides to NPCs demonstrates the potential for these peptides to be used in cell targeting^5,63–65^, especially considering only a sub-population of these cells expressed PSA. For example, PSA-binding peptides may be adapted for selective targeting of cancer cells overexpressing PSA^1,2,13,66^. Hence, the conserved continuous binding pocket of Siglec-11 lent itself to the synergistic study of PSA-binding peptides across various platforms.

Thus, rational peptide design informed on interactions of PSA and the native ligand Siglec-11, supported comparative analysis amongst peptides (including peptides from non-Siglec origins), and provided peptides demonstrating unique applicability. For example, the described design and characterisation strategy saliently revealed the top-performing peptide amongst 762 (I-P24) to originate from the unstructured loop bordering the Siglec binding pocket—thus supporting insight into lectin–PSA interactions while presenting a peptide candidate with desirable affinity and selectivity characteristics across multiple platforms. In this manner, exploiting possible lectin binders to glycans for peptide design can provide lectin-mimics with desirable properties, even if direct experimental study of lectin structure and binding proves challenging. Furthermore, based on the general epitope required for peptide–PSA affinity and selectivity, further investigations may isolate PSA-binding regions of other important proteins thought to interact with PSA (*e.g*., neurotrophins). In future work, hypothesis-driven approaches can build on knowledge of PSA–protein interactions to investigate structural and thermodynamic/kinetic aspects of PSA interactions, improve upon native ligand-based peptide design for polysaccharides, and use design rules from peptide sequence analysis for controlled modulation of ligand affinity and selectivity.

## Methods

### Peptide microarray screening

Peptide library synthesis, microarray preparation, and microarray screening were performed as described previously^25^ with the following specifications. Siglec-related sequences were N-terminally acetylated to match the charge of the corresponding protein fragment. All peptides were printed in triplicate (60 nL spots), and each initial affinity screening was carried out at least three times. Screening was conducted with 10 μM colominic acid (CA; 5 mL per microarray), the *Escherichia coli* homolog of α-2,8-PSA identical in structure to human-derived PSA^67^, and with antibody-based chemiluminescent detection of CA. For selectivity screening, microarrays were challenged with 10 μM CA with 0, 0.1, 1, and 10 μM α-2,8/9-PSA in separate conditions (5 mL total volume each), where molar concentrations were based on molecular weight estimation of synthesized 2,8/9-PSA (details on α-2,8/9-PSA production, purification, and analysis are provided in Supplementary Information). Selectivity screening with monomeric sialic acid was conducted with mass and molar equivalents of N-acetylneuraminic acid (Rose Scientific, Ltd.) along with 10 μM CA (5 mL total volume each). The mass equivalent amount of sialic acid (specifically, 970 μM sialic acid for a given volume of 10 μM CA) was calculated for CA of an average degree of polymerization of 100. For study of pH-dependent binding, affinity screening with CA was performed using PBS buffers of pH 6.0, 7.0, 7.4, and 8.0 (10 mM phosphate, 100 mM NaCl). The impact of NaCl on peptide binding was assessed at pH 7.4 with 0, 100, and 200 mM NaCl. Control dot blot assays for all alternative microarray conditions (*e.g*., α-8/9-PSA competition or pH 6.0) confirmed that antibody detection of CA was not considerably altered in these conditions and that antibodies did not show cross-reactivity with competitors.

Microarray image and data analysis was performed as described^25^. Image transform values for normalization within the image acquisition software were selected based on relative maximum and minimum intensities across all images and hence differed from values selected in previous work; this results in different absolute intensities reported for peptides II-1–II-223, I-P264, and I-P342, though relative intensities are the same (within expected error)^25^. Of the triplicate experiments used to generate mean microarray affinity intensities in standard conditions (within each of which 762 peptides were represented in triplicate), 225 out of 762 values in one of these three experiments were derived from raw data previously reported^25^, but were alternatively analysed as stated. Peptide isoelectric points were calculated with the EMBOSS iep program^68^.

For compositional and positional analyses, residue occurrence within peptide sequences and at each position within sequences was compared between the compiled peptide library and the top 5% affinity binders, as well as between the library and approximately the top 5% selective binders (consisting of peptides with selectivity >65%). For identification of the top 5% selective binders, an affinity constraint was first applied, where peptides displaying background or non-binding intensities (approximately 75% of the library with intensities in the bottom 20% of the intensity range) were excluded along with an additional 5% of very weakly binding peptides (including peptides with intensities in the bottom 30% of the intensity range). Following application of the affinity criteria, 115 peptides (approximately 15% of the library) with binding above background were considered for selectivity assessment. Though select sequences were chosen for modification in the process of peptide design, each set of modified sequences (from different origins) comprised <10% of all sequences and thus inclusion in affinity and selectivity compositional and positional analyses was not considered to affect statistical outcomes through sampling bias.

### Fluorescence anisotropy assay

Peptides (Table 2) were synthesized at >95% purity (confirmed with HPLC) by Biomatik (see Supplementary Information). All peptides were N-terminally acetylated and C-terminally labelled with tetramethylrhodamine dye (TAMRA). TAMRA was chosen as a label due to its pH insensitivity compared to FAM (fluorescein) and related dyes commonly used for fluorescence assays. Experiments were conducted in black 384-well plates with non-binding surface (NBS, Corning, Inc.) to minimize background binding and enhance signal-to-noise ratio in the fluorescence-based binding assay. Titrations were carried out through addition of TAMRA-labelled peptide (8 μL, final concentration 20 nM) to CA (72 μL, final concentrations 0–1500 μM) in PBS, pH 7.4 (10 mM phosphate, 100 mM NaCl), and solutions were incubated with agitation to equilibrium at room temperature (3 h, ~20 °C). Parallel and perpendicular intensities were subsequently measured at 530 nm excitation (band width 5 nm) and 580 nm emission (band width 20 nm) in fluorescence polarization mode with a Tecan Infinite M1000 Pro plate reader. Total fluorescence intensities were also measured as a control for fluorophore properties with target binding^69^; fluorescence intensities of free peptide were determined to be approximately equivalent to intensities with peptide–CA mixtures. At higher concentrations of free CA (control with no peptide addition), attenuation of detected light differed in parallel and perpendicular planes, so respective intensities were subtracted from peptide–CA intensities prior to anisotropy calculations. Increased fluorescence output at high CA concentrations corresponded to higher solution viscosity and likely resulted in light scattering that considerably affected anisotropies without this correction (rheology studies on CA viscosity are provided in Supplementary Information). Unique samples were screened in at least triplicate within each titration experiment, and each peptide titration was carried out at least twice. In experimental repeats, peptide locations on plates were varied to prevent location-specific effects, if any, and different combinations of peptides were assessed at once. Anisotropies were calculated from corrected parallel and perpendicular intensities^69^, and values were fit to the four-parameter logistic binding model^70^ in MATLAB. Prior to curve fitting, anisotropy values for each peptide were transformed through subtraction of minimum values to enhance comparison between peptides. The resulting translations in binding curves were identical, within error, to change in anisotropy curves obtained through subtraction of anisotropy of free labelled peptide. B_max_ and *K*_D_ values obtained were used in the calculation of B_max_/*K*_D_ ratios for comparison to microarray intensity values, and the Pearson correlation coefficient (r) was determined.

### Molecular dynamics simulations

The N-terminal binding domain of Siglec-11 (residues 28–156) was modelled from homologous members of the Siglec family of sialic acid-binding lectins^23,27^ using homology modelling in YASARA as previously described^71–73^. Models for peptides were obtained from the PEP-FOLD web server^74^. Siglec-11 and peptides were equilibrated under all-atom molecular dynamics (MD) at 300 K for 300 ns using the GROMACS engine^75^. All atoms were described using the CHARMM36m forcefield^76^, and solutes were solvated in TIP3P water^77^ and 100 mM NaCl counterions. Equilibrated structures of the peptides were clustered using gmx cluster utility, and the cluster with the highest population was utilized for binding studies. Decasialic acid structures (with either 2,8 or 2,8/9 linkages) were built using the oligosaccharide builder in YASARA. The binding sites for PSA on Siglec-11 were inferred from co-solvent MD simulations where one PSA chain was equilibrated with one Siglec-11 monomer (1:1). Similarly, one peptide was equilibrated with one PSA chain (1:1). In both cases, simulations were carried out in triplicates. Simulations were sampled for 500 ns for Siglec-11 and 250 ns for the peptides. The longest contacting peptide–PSA conformations were isolated for further analysis. Potential of mean force (PMF) calculations were carried out for peptide interactions with PSA via umbrella sampling simulation using GROMACS and PLUMED^78^. The PMF was calculated using weighted histogram analysis. Python packages MDAnalysis^79,80^ and GromacsWrapper were used to build in-house analysis and plotting scripts.

### Cell binding studies

ReNcell VM human neural progenitor cells (NPCs; EMD Millipore) were cultured in accordance with manufacturer’s instructions and passaged at 80–90% confluency with accutase. Immunocytochemistry/immunofluorescence of NPCs with anti-PSA-NCAM antibody (EMD Millipore) was used to confirm PSA expression, as expression varies amongst cell types/populations and decreases with differentiation. NPCs were fixed with methanol prior to blocking with 5% BSA and incubation with anti-PSA-NCAM (EMD Millipore) and Alexa Fluor-labelled goat anti-mouse IgM (Invitrogen).

NPCs were labelled with CellTracker Red CMTPX dye (Thermo Fisher Scientific; 10 μM). Peptide microarray experiments were carried out similarly to carbohydrate screens, except peptide positions were randomized, and microarrays were blocked overnight at 4 °C in 5% BSA (10 mL each) prior to incubation with labelled cells at 37 °C (2 mL on slide surface; 0, 1, 2.5, and 5 million cells). Washes were conducted with PBS, pH 7.4 (3×10 minutes, 10 mL each). Microarrays were air-dried overnight before fluorescence imaging (GE Typhoon Trio+ flatbed scanner; 633 nm excitation, 670BP30 nm emission, 450 PMT, 10 μm resolution). Fluorescence values for cell binding were adjusted by subtraction of control slide intensities (without cells) to account for autofluorescence and non-specific binding. Since background and dynamic range were different with variation in cell numbers, binding ranks were determined to enable comparison of peptide binding between conditions, and the Spearman rank-order correlation coefficient (ρ) was calculated (n = 59).

## Supplementary information

Supplementary Information 1.

Supplementary Information 2.

## Data Availability

Any data generated or analysed during this study that are not included in the published article and Supplementary Information, including raw microarray images and PDB files generated from MD simulations, are available from the corresponding authors upon reasonable request.
