# Supplementary material for: Rational identification and characterisation of peptide ligands for targeting polysialic acid
Source: Sci Rep. 2020 May 6;10:7697. doi: 10.1038/s41598-020-64088-z (PMC7203153; doi:10.1038/s41598-020-64088-z)
Supplement: Supplementary file 1 — Supplementary Information 1. [file 41598_2020_64088_MOESM1_ESM.pdf]

Supplementary Information

Rational identification and characterisation of peptide ligands for targeting polysialic acid

Divya G. Shastry[1,2]*, Flaviyan Jerome Irudayanathan[3], Asher Williams[4], Mattheos Koffas[2,4], Robert J. Linhardt[2,4,5,6], Shikha Nangia[3], and Pankaj Karande[2,4]*

[1] Department of Biological Sciences, Rensselaer Polytechnic Institute, Troy, NY 12180, USA.

[2] Center for Biotechnology and Interdisciplinary Studies, Rensselaer Polytechnic Institute, Troy, NY 12180, USA.

[3] Department of Biomedical and Chemical Engineering, Syracuse University, Syracuse, NY 13244, USA.

[4] Howard P. Isermann Department of Chemical and Biological Engineering, Rensselaer Polytechnic Institute, Troy, NY 12180, USA.

[5] Department of Chemistry and Chemical Biology, Rensselaer Polytechnic Institute, Troy, NY 12180, USA.

[6] Department of Biomedical Engineering, Rensselaer Polytechnic Institute, Troy, NY 12180, USA.

* Email: shastd3@gmail.com (DGS); karanp@rpi.edu (PK)

Contents

S1. Supplementary peptide affinity and selectivity data S4

Figure S1. Relationship of peptide origin to peptide binding on microarray and peptide net charge at pH 7.4 S4

Table S1. *p* values for *z* test for comparison of peptide residue composition between the peptide library and high affinity or high selectivity sequences S5

Table S2. Non-adjusted anisotropy values along with experimental inter-assay standard deviations for peptide–PSA binding in FA assays S6

Table S3. Selectivity of high affinity peptides and peptides with selectivity >80% S7

Figure S2. Microarray binding of FA-tested peptides, and of I-P24, II-P79, and contiguous sequences, to α-2,8-PSA in the presence of different concentrations of α-2,8/9-PSA S9
Figure S3. Positional occurrence of 20 amino acids at positions 1–15 within peptide sequences S10
Table S4. Comparison of sequences with highest probability of occurrence in the compiled peptide library amongst high affinity and high selectivity peptides S11
Figure S4. FA assays for binding of I-P24 and I-P264 with Neu5Ac and CS-A S12
Figure S5. Microarray binding of FA-tested peptides and histidine-containing peptides derived from Siglec-11 to PSA at different pH values S14
S2. Supplementary MD simulations S15
Figure S6. Interactions of I-P24 and II-P336 with α-2,8/9-PSA (decasialic acid) (1:1) as assessed by MD simulations S16
Figure S7. Interaction map showing the normalized number of contacts observed in the MD simulation between Siglec-11 Domain 1 and octasialic acid S18
S3. Production, purification, and analysis of α-2,8/9-PSA S19
Figure S8. ^{1}H NMR spectra of α-2,8/9-polysialic acid obtained from *E. coli* K92 fermentation and of colominic acid S20
Figure S9. TOCSY NMR spectrum of α-2,8/9-polysialic acid S21
Figure S10. TBE-PAGE of glycans for molecular weight estimation of *E. coli*-derived α-2,8/9-PSA S22
S4. Quality control of TAMRA-labelled peptides S23
Figure S11. Mass spectrometry report for I-P2 S23
Figure S12. HPLC report for I-P2 S24
Figure S13. Mass spectrometry report for I-P24 S25
Figure S14. HPLC report for I-P24 S26
Figure S15. Mass spectrometry report for I-P25 S27
Figure S16. HPLC report for I-P25 S28
Figure S17. Mass spectrometry report for I-P264 S29
Figure S18. HPLC report for I-P264 S30

Figure S19. Mass spectrometry report for II-P76 S31
Figure S20. HPLC report for II-P76 S32
Figure S21. Mass spectrometry report for II-P79 S33
Figure S22. HPLC report for II-P79 S34
Figure S23. Mass spectrometry report for II-P104 S35
Figure S24. HPLC report for II-P104 S36
Figure S25. Mass spectrometry report for II-P336 S37
Figure S26. HPLC report for II-P336 S38
Figure S27. Mass spectrometry report for II-P341 S39
Figure S28. HPLC report for II-P341 S40
S5. Rheology for colominic acid viscosity assessment S41
Figure S29. Change in CA solution viscosity with concentration S41
S6. References for supplementary information S42

S1. Supplementary peptide affinity and selectivity data

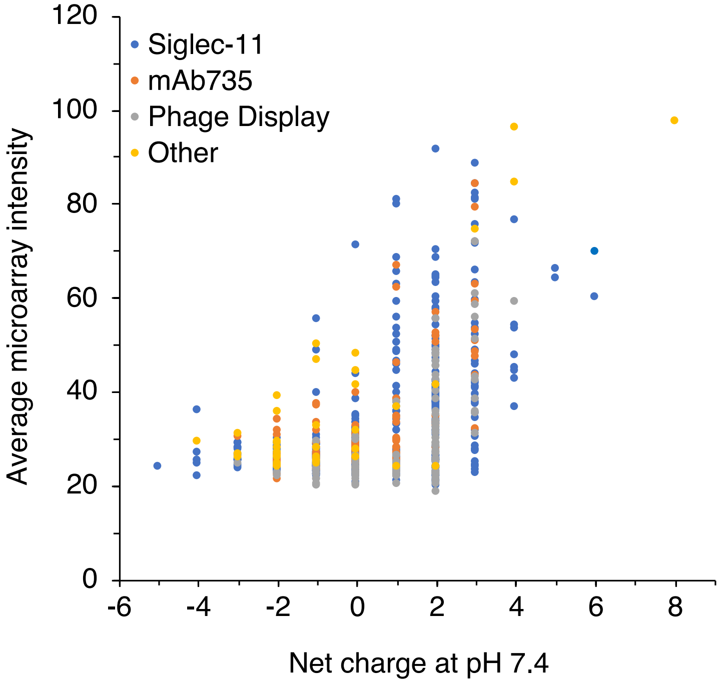

Supplementary Figure S1. Relationship of peptide origin to peptide binding on microarray and peptide net charge at pH 7.4. "Siglec-11," "mAb735," and "Phage Display" indicate peptides derived from these sources directly as well as modified sequences from these sources. "Other" includes peptides from literature,[13] random sequences, and *de novo* designed sequences. All mAb and phage display peptides, along with two Siglec-11-derived peptides, are from Shastry and Karande.[12] Binding intensities represent the mean of three independent experiments, with triplicate intra-assay measurements (pooled inter-assay standard deviation for 762 peptides = 8.3; error bars excluded for clarity). The independence of peptide affinity on peptide origin may be due to the limited sequences screened and exemplary peptide origins; though high-throughput screening was employed, larger screens of ~10,000 peptides or greater may be required to extract origin-based information on binding.

Residue/residue type	Top 5% affinity	Top 5% selectivity
D	N/A	0.0001
E	0.0000	0.0000
R	0.0000	0.0000
K	0.0000	0.0000
H	N/A	0.3829
C	N/A	N/A
N	0.0000	0.0000
Q	N/A	0.0000
S	0.0000	0.0000
T	0.0000	0.0000
Y	N/A	0.0003
F	0.0000	0.0001
W	N/A	N/A
G	0.0000	0.0000
A	0.0865	0.0000
V	0.8244	0.0000
I	N/A	0.0408
L	0.0000	0.0003
M	N/A	N/A
P	0.0358	0.0000
Acidic	0.0000	0.0000
Basic	0.0000	0.0000
Polar	0.0000	0.0000
Aromatic	0.0000	0.0000
Nonpolar	0.0516	0.0000

Supplementary Table S1. *p* values for *z* test for comparison of peptide residue composition between the peptide library and high affinity or high selectivity sequences (corresponding to Fig. 2). Red = statistically significant decrease, green = statistically significant increase, and yellow = not statistically significant.

PSA concentration (μM)	I-P2	I-P24	I-P25	I-P264	II-P76	II-P79	II-P104	II-P336	II-P341
0.00	37.6 ± 0.3	28.4 ± 1.9	24.9 ± 0.2	28.5 ± 1.3	42.7 ± 1.3	48.4 ± 0.3	31.3 ± 2.2	29.7 ± 0.6	33.1 ± 1.0
0.15	33.8 ± 2.3	26.9 ± 1.0	24.1 ± 1.0	26.5 ± 0.7	40.1 ± 0.1	46.5 ± 0.1	29.1 ± 1.2	26.8 ± 0.8	31.9 ± 0.4
1.50	32.9 ± 1.8	26.7 ± 0.3	22.8 ± 0.5	25.7 ± 0.9	39.0 ± 0.3	45.1 ± 0.1	27.8 ± 1.3	26.3 ± 1.0	30.8 ± 0.6
15.0	33.3 ± 3.0	27.1 ± 0.2	23.5 ± 1.3	26.0 ± 1.3	39.9 ± 0.6	46.7 ± 1.2	28.7 ± 1.8	28.2 ± 1.3	30.9 ± 0.5
150	33.2 ± 3.8	36.3 ± 0.5	23.8 ± 1.3	26.9 ± 2.1	42.7 ± 0.1	49.9 ± 0.4	33.0 ± 2.5	34.4 ± 1.4	32.5 ± 1.0
500	35.5 ± 4.9	48.4 ± 1.1	26.6 ± 3.0	28.6 ± 2.5	48.6 ± 0.2	56.1 ± 0.8	39.4 ± 3.3	42.9 ± 0.2	36.0 ± 1.3
1000	35.6 ± 9.5	56.1 ± 2.4	28.5 ± 5.2	30.9 ± 4.8	54.9 ± 0.0	59.9 ± 0.1	43.4 ± 4.8	48.9 ± 0.6	41.2 ± 3.9
1500	35.8 ± 11.5	60.4 ± 4.8	29.4 ± 8.2	33.0 ± 7.8	58.7 ± 0.1	63.4 ± 0.9	44.6 ± 8.0	51.3 ± 1.0	44.6 ± 4.2

Supplementary Table S2. Non-adjusted anisotropy values along with experimental inter-assay standard deviations for peptide–PSA binding in FA assays. Errors are standard deviations of the mean from at least two independent experiments, with at least triplicate measurements within each assay.

Peptide index	Peptide sequence	Peptide origin	% selectivity
**(a) Selectivity of high affinity peptides**			
I-P23	YWFKGRTSPKTGAPV	Siglec-11	43 ± 3
I-P24*	FKGRTSPKTGAPVAT	Siglec-11	85 ± 10
I-P59	LSNAFFLKVTALTKK	Siglec-11	52 ± 10
I-P60	NAFFLKVTALTKKPD	Siglec-11	70 ± 18
I-P62	LKVTALTKKPDVYIP	Siglec-11	67 ± 17
I-P85	AALSPRRTRPSTSHF	Siglec-11	59 ± 5
I-P86	LSPRRTRPSTSHFSV	Siglec-11	62 ± 10
I-P102	VDFSRKGVSAQRTVR	Siglec-11	59 ± 10
I-P103	FSRKGVSAQRTVRLR	Siglec-11	55 ± 11
I-P104	RKGVSAQRTVRLRVA	Siglec-11	45 ± 4
I-P143	WGPRTLGLELRGVRA	Siglec-11	37 ± 1
I-P278	FRVKICRKEARKRAA	Siglec-11	43 ± 8
I-P279	VKICRKEARKRAAAE	Siglec-11	46 ± 12
I-P330	WFKGRTSPKTGAPVA	Siglec-11	35 ± 1
I-P333	WFKG**K**TSPKTGAPVA	Siglec-11: R5K mutation	59 ± 2
I-P378	KGKGKGKGKGKGKGK	*De novo*	60 ± 9
I-P379	KGGGKGGGKGGGKGG	*De novo*	61 ± 7
I-P380	GGKGGGKGGGKGGGK	*De novo*	56 ± 7
II-P35	GSGSGTDFTLKISRV	mAb735	53 ± 6
II-P50	VPYTFGGGTRLEIKG	mAb735	71 ± 9
II-P77	PGSGNTKYNEKFKGK	mAb735	49 ± 5
II-P79	NTKYNEKFKGKATLT	mAb735	52 ± 11
II-P213	AISSPLL**R**NPFRGGGS	Phage display screening: W8R mutation	66 ± 9
II-P214*	AISSPLL**K**NPFRGGGS	Phage display screening: W8K mutation	80 ± 13
II-P336*	NRTVL**R**NLGNGTSLP	Siglec-11: E6R mutation	81 ± 12
**(b) High selectivity peptides**			
I-P24	FKGRTSPKTGAPVAT	Siglec-11	85 ± 10
I-P25	GRTSPKTGAPVATNN	Siglec-11	83 ± 13
I-P307	QELHYASLSFQGLRL	Siglec-11	80 ± 14
I-P321	YSEIKIHTGQPLRGP	Siglec-11	85 ± 18
I-P323	KIHTGQPLRGPGFGL	Siglec-11	86 ± 8
I-P344	GRTS**A**KTGAPVATNN	Siglec-11: P5A mutation	85 ± 8
I-P346	GRTSPK**A**GAPVATNN	Siglec-11: T7A mutation	91 ± 15
II-P145	TLPAILQ**A**SGTRGGGS	Phage display screening: S8A mutation	81 ± 16
II-P214	AISSPLL**K**NPFRGGGS	Phage display screening: W8K mutation	80 ± 13

II-P336	NRTVL**R**NLGNGTSLP	Siglec-11: E6R mutation	81 ± 12

Supplementary Table S3. **(a)** Selectivity of high affinity peptides **(b)** Peptides with selectivity >80% and their corresponding selectivities. High affinity peptides exhibit binding intensities in the top 5% in three independent screens, with triplicate measurements within each screen and inter-assay coefficients of variation <25% (peptides exhibiting intensities in the top 5% with higher inter-assay CVs excluded). Similarly, only high selectivity peptides with <25% coefficients of variation in affinity screening are displayed. Selectivity error represents standard deviations of the mean of triplicate intra-assay measurements. Bolded residues represent mutations from parent peptides. * High affinity peptides with selectivity >80%.

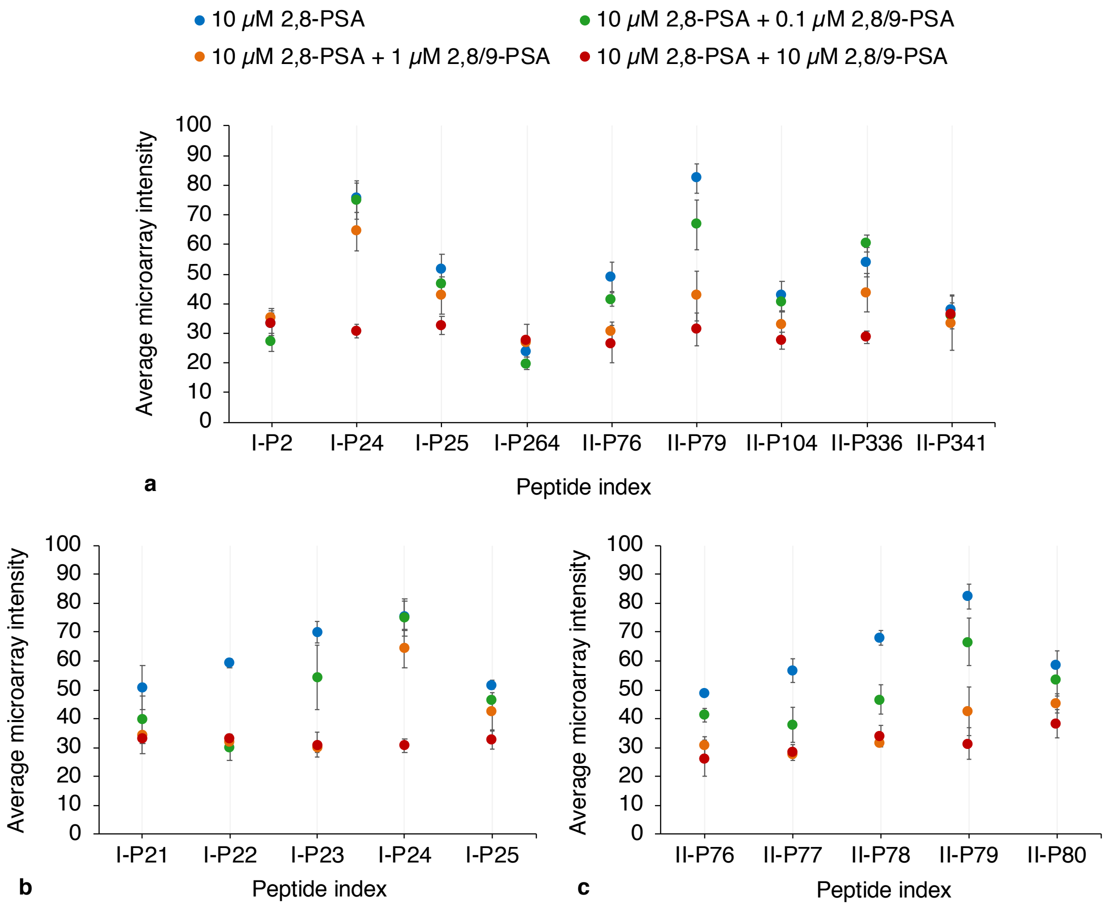

Supplementary Figure S2. **(a)** Microarray binding of FA-tested peptides to α-2,8-PSA in the presence of different concentrations of α-2,8/9-PSA. Of these peptides, I-P24 emerges as the top PSA-binding peptide with regard to both affinity and selectivity. **(b–c)** Microarray binding of I-P24 **(b)** and II-P79 **(c)** and contiguous sequences to α-2,8-PSA in the presence of different concentrations of α-2,8/9-PSA. Adjacent peptides are shifted by two amino acids. Peptides immediately beyond displayed ranges do not exhibit PSA binding. Note that I-P24 and I-P25, as well as II-P76 and II-P79, show similar K_D values in the FA assay, while II-P76 and II-P79 show similar B_{max} values. Thus, the differing "affinity" responses of these peptide pairs in microarray screening agrees with response differences seen for I-P24 and I-P25 in FA, but does not match response differences observed for II-P76 and II-P79 (for which microarray intensities vary but anisotropy responses by concentration are similar); however, both II-P76 and II-P79 show generally high microarray binding, and differences from FA results possibly arise from microarray error and peptide immobilization effects, including density effects. Error bars represent standard deviations of the mean of triplicate measurements.

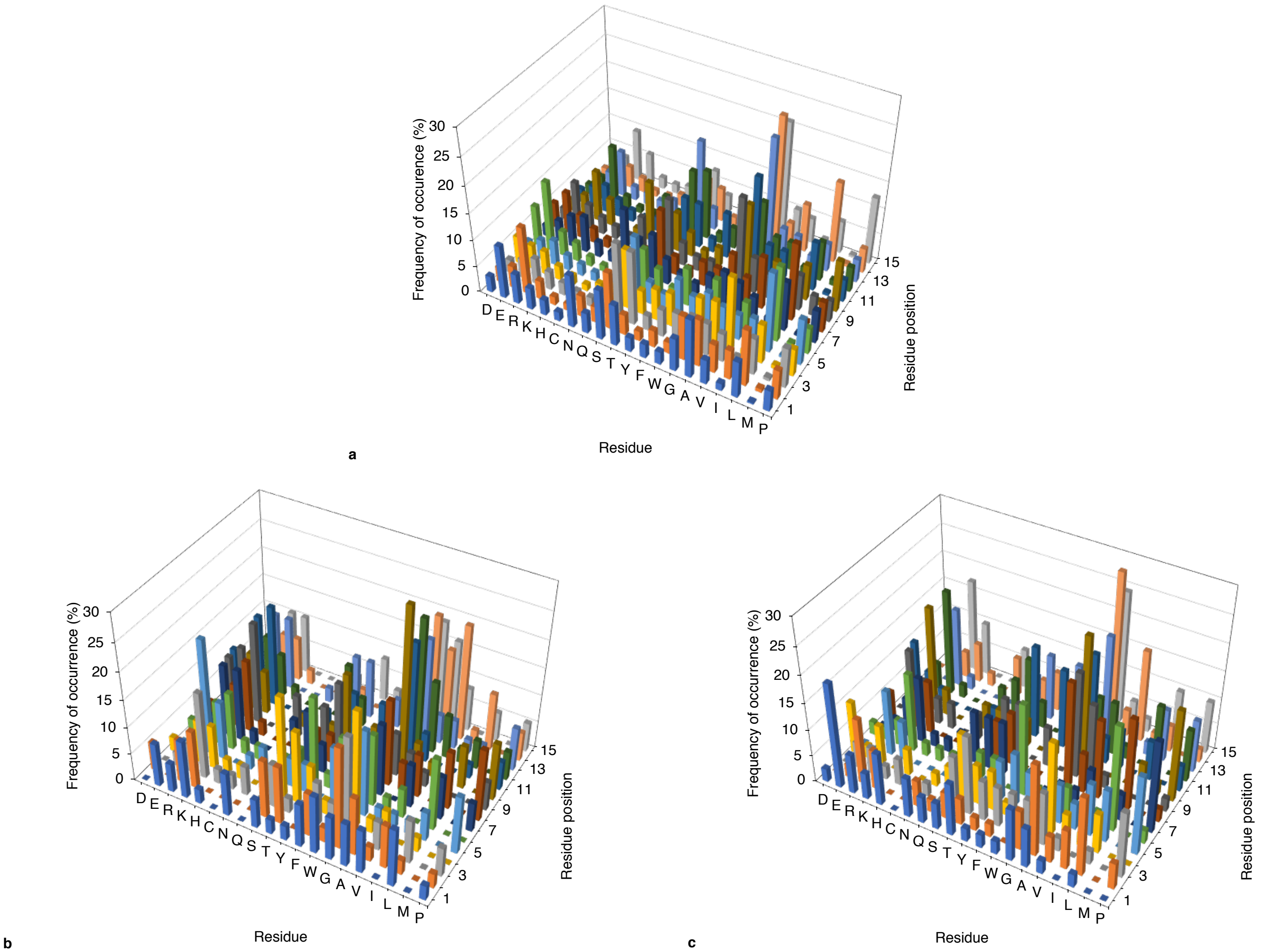

Supplementary Figure S3. Positional occurrence of 20 amino acids at positions 1–15 within peptide sequences. Position 16 excluded for clarity (only phage display-derived peptides contained a residue at position 16 [15 Ser and 1 Gly]). **(a)** Compiled peptide library **(b)** Top 5% affinity peptides **(c)** Top 5% selective peptides. Statistical significance of changes in positional occurrences was not determined as low residue occurrence in the sample populations precluded the assumption of normal distribution.

Residue position (N to C)	1	2	3	4	5	6	7	8	9	10	11	12	13	14	15	16
**Peptide library**	A	R	S	V	L	L	N	L	G	G	G	T	G	G	G	S
**Top 5% affinity peptides**	K/ W/ L	G	G	G	R	T	R/ K/ G	G	K	G	G	G	G	G	G	S
**Top 5% selective peptides**	E	A	T	A	P	L	P	G	G	G	G	R	G	G	G	S

Supplementary Table S4. Comparison of sequences with highest probability of occurrence in the compiled peptide library amongst high affinity peptides (top 5%) and amongst high selectivity peptides (top 5%).

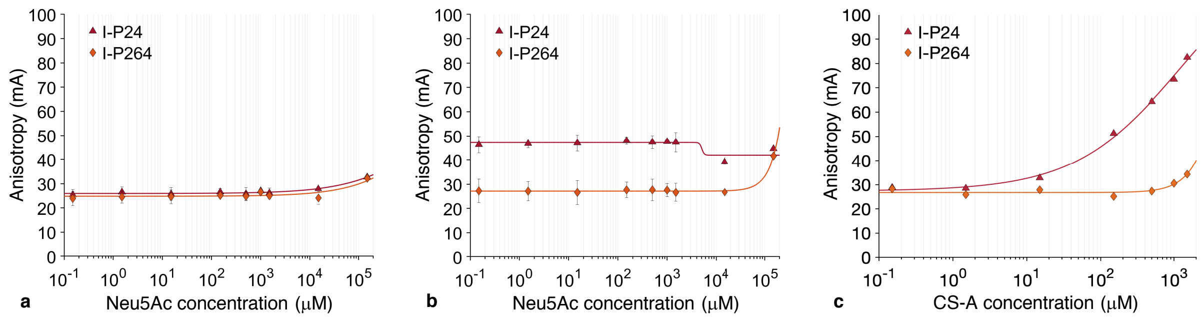

Supplementary Figure S4. **(a–b)** Interaction of I-P24 and I-P264 with Neu5Ac as assessed by fluorescence anisotropy (FA) titrations in the **(a)** absence or **(b)** presence of 500 μM PSA. For a given volume, the highest concentration tested (150 mM) is approximately mass equivalent to 1.5 mM PSA. I-P24 shows minimal binding to Neu5Ac, even at higher concentrations. In the competitive FA assay, binding of I-P24 to PSA is largely maintained, except for a small decrease in anisotropy at the highest concentrations of Neu5Ac, indicating specific peptide binding to PSA. Error bars represent standard deviations from two independent experiments (0.15–15000 μM), with triplicate intra-assay measurements (0.15–150000 μM). **(c)** FA assay for binding of I-P24 and I-P264 to chondroitin sulphate A (CS-A). CS-A was used instead of α-2,8/9-PSA due to higher purity of the commercially available glycan (for accurate assessment with FA, high purity reagents must be used). K_D for I-P24 binding to CS-A was determined to be higher than for PSA at >1 mM; as with α-2,8/9-PSA microarray selectivity experiments, microarray studies with CS-A competition to Siglec-derived peptides identified I-P24 as PSA-selective. Anisotropies were determined from triplicate measurements (error bars excluded for clarity). FA assays with Neu5Ac and CS-A were conducted as described for peptide–PSA affinity studies, except Neu5Ac concentrations up to 150 mM were used. Also, for competitive Neu5Ac and PSA binding, TAMRA-labelled peptide was added to CA (45 μL, final concentration 500 μM) and Neu5Ac (27 μL, final concentrations 0–150 mM) in PBS, pH 7.4.

Assessment of selective binding of peptides with the FA assay is only presented in the context of alternative glycans as proof-of-principle above. However, given the comparatively lower sensitivity of microarray screening, FA may be employed for quantifying affinity in various buffers to assess the effects of various ions, if any, on PSA binding. Many carbohydrates and polyelectrolytes are known to be influenced by solution ions in ligand binding,[1–5] and given the

influence of various metal ions on PSA conformation and binding,[6,7] it is possible PSA–peptide binding can be modulated by solution components. The FA assay serves as a flexible tool for conducting such binding analyses, which would provide critical information when PSA-binding peptides are applied in *in vitro* or *in vivo* settings (for example, in complex cell media).

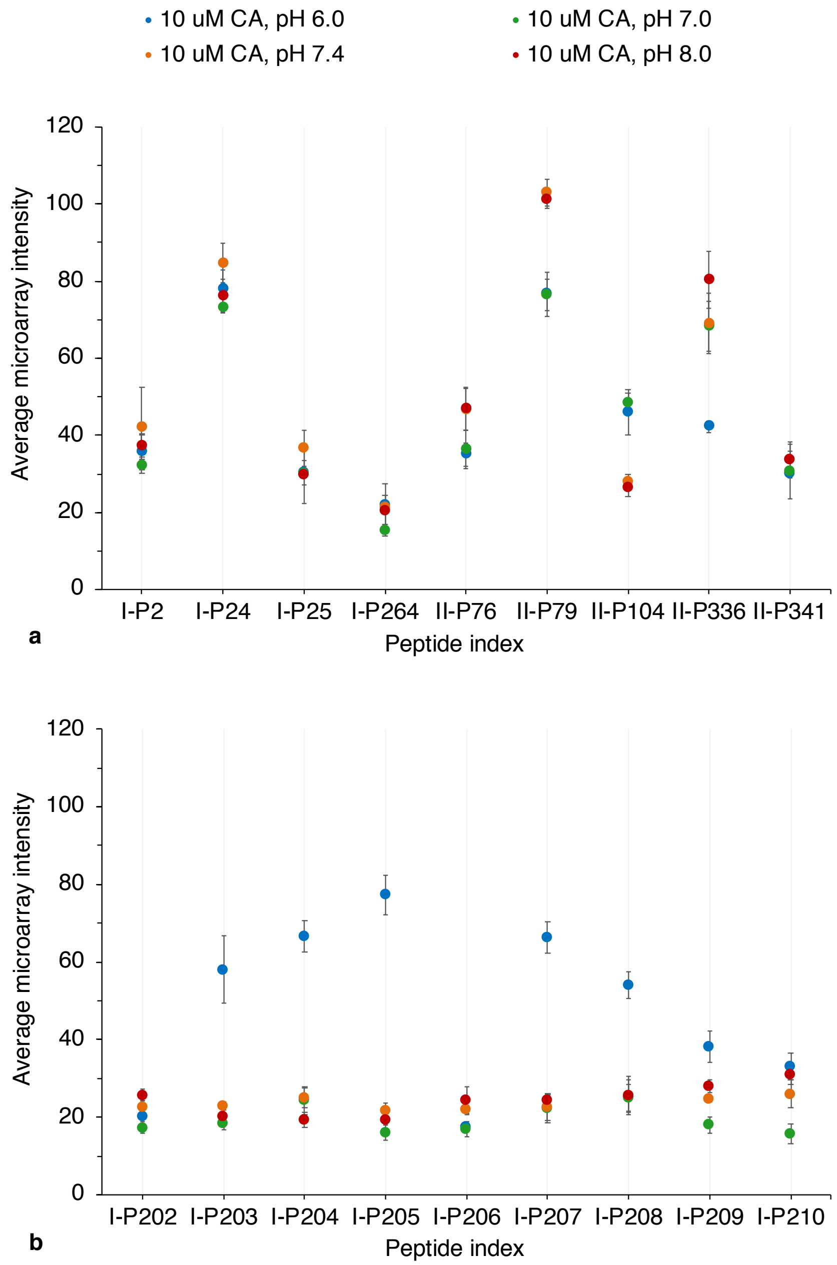

Supplementary Figure S5. **(a)** Microarray binding of FA-tested peptides to PSA at different pH values. Binding remains unaltered or similar (within error) as compared to binding in the standard pH 7.4 condition. **(b)** Binding of a series of histidine-containing peptides derived from Siglec-11 at different pH values (each peptide is shifted from the next by two amino acids). At pH 6.0, peptides gain positive charge, enhancing interaction with negatively charged PSA. I-P206 is the exception in this trend and may not bind due to sequence specific charge presentation. Error bars represent standard deviations of the mean of triplicate measurements.

S2. Supplementary MD simulations

MD simulations were performed with α-2,8/9-PSA, similarly to simulations with α-2,8-PSA. Simulations suggested some binding of α-2,8/9-PSA to Siglec-11, with direct interaction of the ligand to the protein domain in similar regions as observed in the simulation with 2,8-PSA, including to the Arg and Lys in the CC′ loop. However, binding appeared to demonstrate less preference to the 2,8-PSA binding site, with a lower number of interaction sites on the G strand over the course of the complete simulation trajectory. PSA-binding peptides I-P24 and I-P50 were observed to have higher interaction with α-2,8-PSA as compared to α-2,8/9-PSA (Fig. S6). Specificity differences (for the protein and/or peptides) likely arise from the higher helical pitch of the polymer with alternating glycosidic linkage. In microarray studies, I-P24 bound α-2,8-PSA in the presence of a fraction of the linkage isomer better than most other peptides tested, and simulations showed that I-P24 exhibits both a greater number of interactions/contacts and interaction over a longer time period with α-2,8-PSA. In comparison, II-P336 also showed greater selectivity in microarray studies to 2,8/9-PSA than most other peptides, and simulations did not demonstrate a considerable qualitative difference in interaction of this peptide between 2,8-PSA and 2,8/9-PSA. However, it is important to note that theoretical K_D values were not obtained from these simulations, and the use of decasialic acid (as compared to a higher molecular weight polymer used experimentally) may affect binding affinities.[6–8] As such, these results are not contradictory to microarray selectivity studies where PSA-binding peptides were classified as having higher selectivity to α-2,8-PSA, with classification based on 10% competition (*i.e.*, no affinity constant determination) and relative selectivity ranking of peptides. Thus, selectivity assessment through experimental and computational methods, though both surrogates for direct K_D measurement, revealed similar conclusions on peptide binding. Furthermore, the applicability of the microarray selectivity measure also holds for the purposes of peptide/residue property determination, as demonstrated.

Simulations for both α-2,8- and α-2,8/9-PSAs were additionally carried out with 10:1 ratio of peptide:PSA. As with 1:1 simulations, PSA-binding peptides oriented such that positively charged residues interacted directly with the PSA, with higher-binding peptides clustering closer to the polymer. II-P341, a moderate to weak binder in microarray and FA assays, did not show direct interaction with α-2,8-PSA in 1:1 simulation, but demonstrated binding in 10:1 simulation,

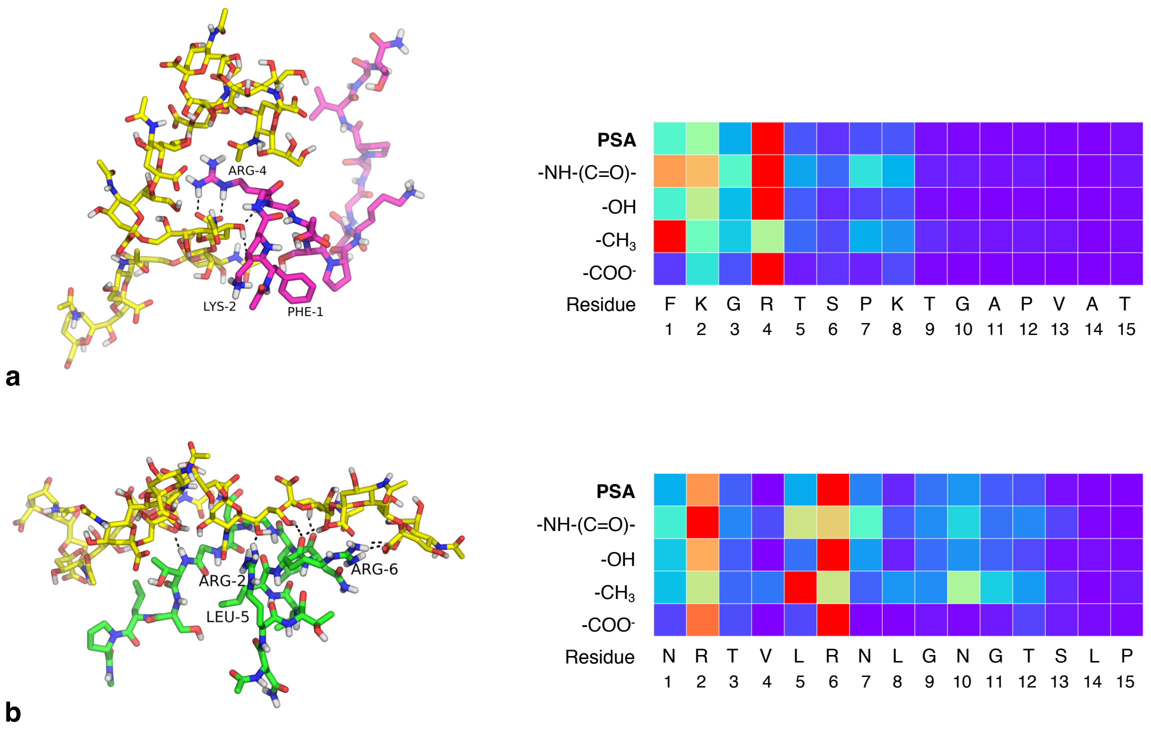

Supplementary Figure S6. Interactions of **(a)** I-P24 and **(b)** II-P336 with α-2,8/9-PSA (decasialic acid) (1:1) as assessed by MD simulations. Simulation frames with the greatest interaction between ligands are displayed (with a cut-off distance for interaction set at 3.0 Å). Polar contacts at the time point of the snapshots are shown as black dashes, and residues for which highest interaction is demonstrated over the course of the complete simulation trajectory are labelled. The carbohydrate is shown in yellow, and peptides I-P24 and II-P336 are shown in magenta and green, respectively. Corresponding residue interaction maps (right) show the normalized number of contacts (3.0 Å cut-off distance) from 0 (purple) to 1 (red) observed over the complete time course of simulation between each peptide and decasialic acid. I-P50, I-P264, and I-P341 did not interact with α-2,8/9-PSA in simulations, and I-P24 showed less interaction with α-2,8/9-PSA over the course of the simulation as compared to simulations with α-2,8-PSA. Simulation snapshots for **(a–b)** as well as for α-2,8/9-PSA with Siglec-11 are provided as Supplementary PDB files.

suggesting that ligand density may play a role in the binding properties of this peptide. Preliminary experimental density studies, where the peptide was immobilized on the microarray at higher

density, also suggested higher binding with higher peptide density. Furthermore, in simulations, II-P341 was more prone to remain in interaction with PSA when its secondary structure was constrained. Thus, II-P341 may represent a case where peptide presentation is especially important for binding and thus presents higher variability amongst the different testing platforms.

10:1 simulations were carried out with chondroitin sulphate A (CS-A) replacing PSA. CS-A was used in the FA assay to demonstrate the comparatively higher binding of I-P24 to α-2,8-PSA, since production of α-2,8/9-PSA of sufficient quantity and purity for the assay was not feasible. The CS-A simulations supported the proof-of-concept experimental FA results, and CS-A was observed to interact to a lesser extent with I-P24 than PSA.

All previously described peptide simulations were performed for the following five peptides: I-P24, I-P50, I-P264, II-P336, and II-P341. 1:1 and 10:1 simulations of an additional eight peptides with α-2,8-PSA demonstrated similar mechanisms of peptide interaction with PSA as for the initial five peptides.

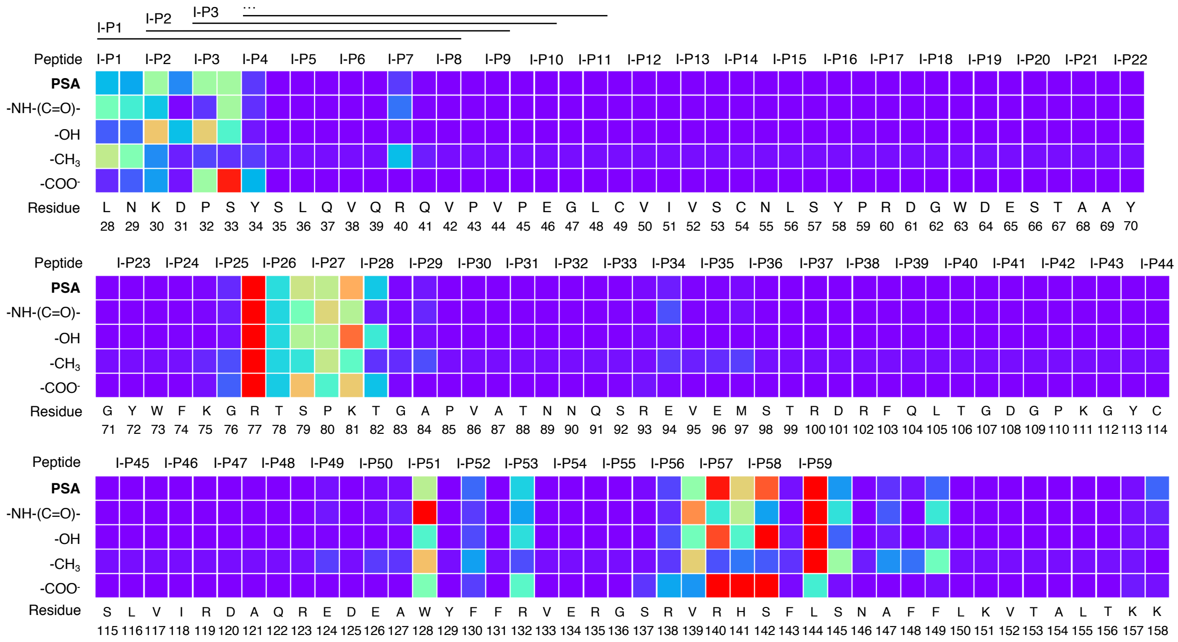

Supplementary Figure S7. Interaction map showing the normalized number of contacts (3.0 Å cut-off distance) from 0 (purple) to 1 (red) observed in the MD simulation between Siglec-11 Domain 1 and octasialic acid. Residues correspond to Siglec-11 numbering, and peptide identifiers mark the start of each 15-residue peptide sequence. Contacts are highly similar to contacts made in simulation of Siglec-11 binding to decasialic acid, where the CC′ loop and F/G strand show prominently higher interaction, and peptides derived from these regions display higher relative binding to NPCs (Fig. 6). Domain 1 residues represented in this simulation include complete N- and C-terminal linker regions (residues 28–30 and residues 135–158, respectively). The terminal portion of the C-terminal linker region is unstructured and does not demonstrate PSA-binding in protein form; hence, the unstructured linker was truncated for Domain 1 simulation with decasialic acid. I-P59 (Table 1 and Fig. 6) likely shows high experimental PSA binding due to the terminal Lys residues.

S3. Production, purification, and analysis of α-2,8/9-PSA

Strains, culture media, and growth conditions. Luria-Bertani (LB) medium was used for overnight cell culture growth. A rich defined medium developed from adapted protocols[9] was used for all the shake flask fermentations (3.5 g/L KH_2PO_4, 5.0 g/L K_2HPO_4, 3.5 g/L $(NH_4)_2HPO_4$, 100 mL of 10x MOPS mixture, 11.3 g/L asparagine [nitrogen source], 1 mL of 1 M $MgSO_4$, 0.1 mL of 1 M $CaCl_2$, and 1 mL of 0.5 g/L thiamine HCL, supplemented with 8.4 g/L xylose as a carbon source; 10x MOPS mixture consisted of 83.7 g/L MOPS, 7.2 g/L tricine, 28 mg/L $FeSO_4 \cdot 7H_2O$, 29.2 g/L NaCl, 5.1 g/L NH_4Cl, 1.1 g/L $MgCl_2$, 0.5 g/L K_2SO_4, and 0.2 mL of micronutrient stock, and micronutrient stock consisted of 0.2 g/L $(NH_4)_6Mo_7O_{24}$, 1.2 g/L H_3BO_3, 0.1 g/L $CuSO_4$, 0.8 g/L $MnCl_2$, and 0.1 g/L $ZnSO_4$). Final pH of the medium was adjusted to 8.5 with 5 M KOH. All nutrients and chemicals for medium preparation were from Sigma Chemical Company.

A colony was picked from an LB agar plate streaked with cells from *E. coli* K92 (ATCC 35860), and a 5 mL pre-culture was grown in LB medium overnight at 37 °C. One millilitre of this pre-culture was transferred to 1 L of rich defined medium in a Pyrex Fernbach culture flask (Corning Life Sciences). The cell cultures were incubated in a rotary air shaker (New Brunswick Scientific Innova 44R) at 37 °C, 250 rpm and allowed to grow for ~76 h until the release of the capsular polysaccharide in the medium was maximal in the stationary phase of growth.[10] Insoluble material and cells were removed by centrifugation at 4 °C (5,000 x *g* for 1 h), and the supernatant fluid was collected and filtered using a 0.45 μm Corning bottle-top vacuum filter. The supernatant was then concentrated to ~10 mL by tangential flow filtration using a Vivaflow 200 cassette (Sartorius) with exclusion size of 10 kD.

Purification of capsular α-2,8/9-PSA. Purification of capsular polysaccharide (CPS) was carried out as follows. The concentrated cell culture supernatant was precipitated with 80% (v/v) cold ethanol and stored at -20 °C overnight. After precipitation, the pellet was collected and re-suspended in DNAse digestion buffer in digestion buffer (100 mM Tris, pH 7.5, 50 mM $MgCl_2$, 10 mM $CaCl_2$) and digested with DNAse (1.5 mg/L; Sigma) by incubation at 37 °C for 1 h followed by Protease K digestion (2 mg/mL; Sigma) incubating at 56 °C for 2 h. After a second ethanol precipitation, the pellet was collected, re-dissolved in water, and filtered through a 10K or 3K spin column to remove residual small peptides and salt. The retentate was extensively dialysed against distilled water and, finally, lyophilised for NMR analysis and further experiments.

α-2,8/9-PSA analysis. The purified CPS from the supernatant was analysed by one-dimensional ^{1}H nuclear magnetic resonance (NMR).[11] NMR experiments were performed on a Bruker Advance II 600 MHz spectrometer (Bruker Bio Spin) with Topsin 2.1.6 software (Bruker). Samples were dissolved in 0.5 mL D_2O (99.96%; Sigma) and freeze-dried repeatedly to remove the exchangeable protons. The samples were re-dissolved in 0.4 mL D_2O and transferred to NMR microtubes (outside diameter 5 mm; Norell). The conditions for one-dimensional ^{1}H NMR spectra were as follows: wobble sweep width of 12.3 kHz, acquisition time of 2.66 s, relaxation delay of 8.00 s, and temperature of 298 K.[9] The NMR spectrum in Fig. S8 was obtained from 32 scans. A spectrum of colominic acid (α-2,8-PSA) from Nacalai USA, Inc. was similarly obtained for comparative purposes. NMR standard spectral data was used to confirm peak assignments and assess product purity.

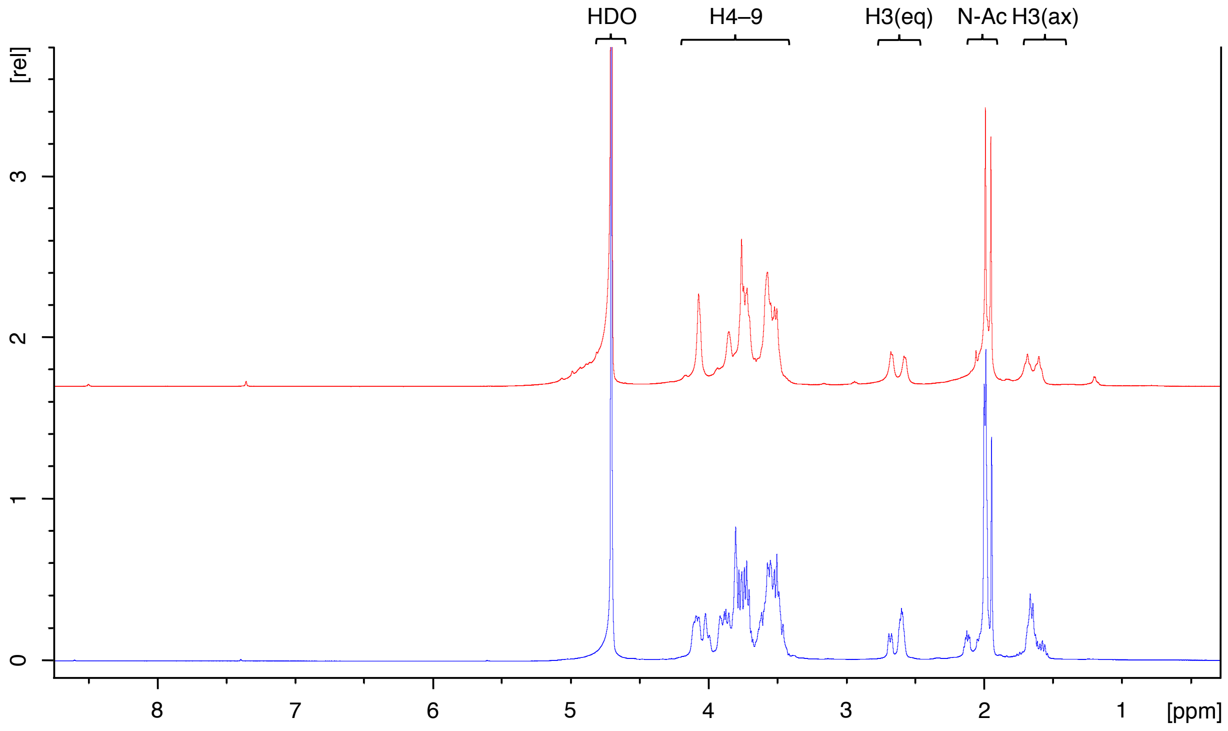

Supplementary Figure S8. ^{1}H NMR spectra of α-2,8/9-polysialic acid obtained from *E. coli* K92 fermentation (top) and of colominic acid from Nacalai USA, Inc. (α-2,8-polysialic acid; bottom). Variation between the spectra may arise from the linkage difference as well differences in the secondary structure of the polymer chains.[14,15]

Two-dimensional total correlation spectroscopy (2D ^1H–^1H TOCSY) was additionally performed to confirm the presence of two glycosidic linkages in the α-2,8/9-PSA. Purified freeze-dried PSA (15–20 mg) was dissolved in 400 µL D_2O (99.96%), lyophilized two times to remove exchangeable protons, and transferred to an NMR tube (diameter 5 mm; Norell). TOCSY was performed on an 800-MHz NMR spectrometer (Bruker) at a temperature of 298 K. The spectrum displayed in Fig. S9 was acquired from 16 scans and using Topsin 2.1.6 software (Bruker).

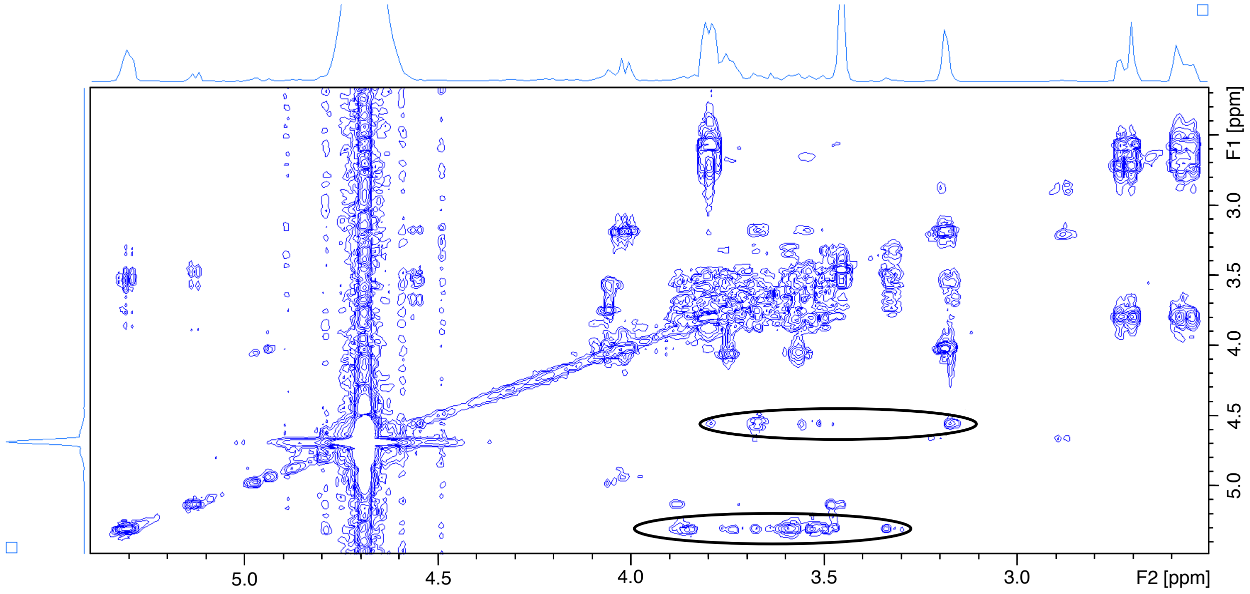

Supplementary Figure S9. TOCSY NMR spectrum of α-2,8/9-polysialic acid. In comparison to the TOCSY spectrum of colominic acid,[16] which contains only α-2,8 linkages, the spectrum of α-2,8/9-PSA contains additional peaks (circled regions) indicating the presence of two glycosidic linkages (*i.e.*, α-2,8 and α-2,9 linkages).

Molecular weight and polydispersity of the *E. coli* derived α-2,8/9-PSA was estimated with TBE-PAGE (Fig. S10). 210 µg and 52.5 µg of colominic acid (Nacalai USA, Inc.; average molecular weight 30 kD), chondroitin sulphate A (CS-A; from bovine trachea, Sigma-Aldrich; average molecular weight estimated at 45 kD[12]), fucoidan (Sigma-Aldrich; average molecular weight 20 kD), and heparin (Sigma-Aldrich; ladder 2.5–10.2 kD) were run on a 4–20% Novex TBE gel (Invitrogen) alongside samples of α-2,8/9-PSA and purification pellet. The gel was stained with alcian blue with image acquisition in grayscale. α-2,8/9-PSA was estimated to have an average molecular weight of 75 kD. As with our previous work,[12] this estimation, while broad, was used to determine relative amounts of competing PSA. As such, comparisons between

microarray selectivity conditions and between peptides hold, though comparative amounts of 2,8-PSA and 2,8/9-PSA include error from this estimate and from polydispersity in molecular weights.

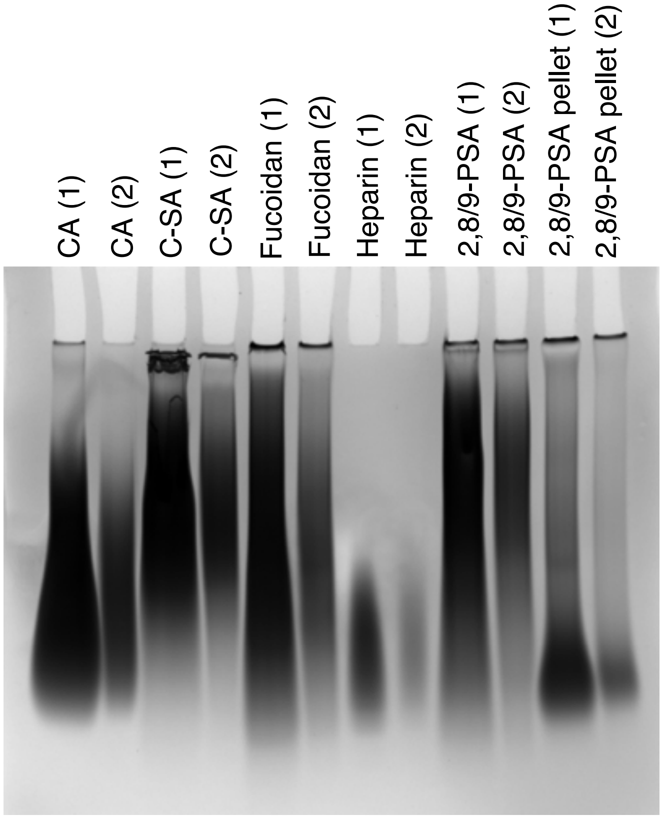

Supplementary Figure S10. TBE-PAGE of glycans for molecular weight estimation of *E. coli*- derived α-2,8/9-PSA. Concentration (1) = 210 µg and concentration (2) = 52.5 µg.

The presence of nucleic acid contamination in purified α-2,8/9-PSA was assessed with UV-Vis spectrophotometry (NanoDrop 1000, Thermo Fisher Scientific). In the UV-Vis spectrum of product samples, concentration-dependent absorbance at ~230 nm from the sialic acid amide group was evident, and 260 nm absorption suggested the potential presence of nucleic acid impurities. Quantification of DNA with PicoGreen (Quant-IT PicoGreen dsDNA Assay, Thermo Fisher Scientific) indicated that DNA was present at <0.4%. Though the assay is most sensitive for dsDNA (among various nucleic acids), and the possibility of assay interference by contaminants or α-2,8/9-PSA itself exists, the product was considered suitable for use in microarray screenings, as NMR data also demonstrated absence of major impurities.

S4. Quality control of TAMRA-labelled peptides

Quality control of TAMRA-labelled peptides (used in FA assays) was performed by Biomatik. Mass spectral analysis confirmed molecular weight specifications for synthesized peptides, and purity of all peptides was confirmed to be >95% with HPLC.

Mass Spectrometry Report

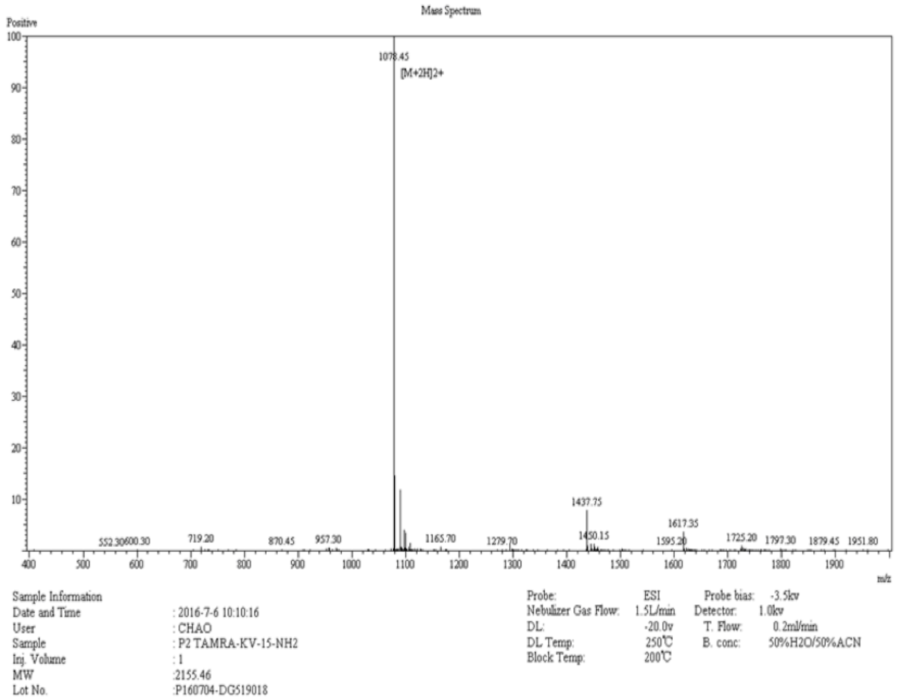

Supplementary Figure S11. Mass spectrometry report for I-P2 (TAMRA-KDPSYSLQVQRQVPV-NH_2). MW = 2155.46 Da.

HPLC Report

Structure : P2 TAMRA-KV-15-NH2
Lot No : P160704-DG519018
Column : 4.6×250mm,Venusil MP C18-5
Solvent A : 0.1% trifluoroacetic in 100% acetonitrile
Solvent B : 0.1% trifluoroacetic in 100% water
Gradient :

	A	B
0.01min	22%	78%
25min	47%	53%
25.1min	100%	0%
30min	STOP	

Flow rate : 1.0 mL/min
Wavelength : 220nm
Volume : 5ul

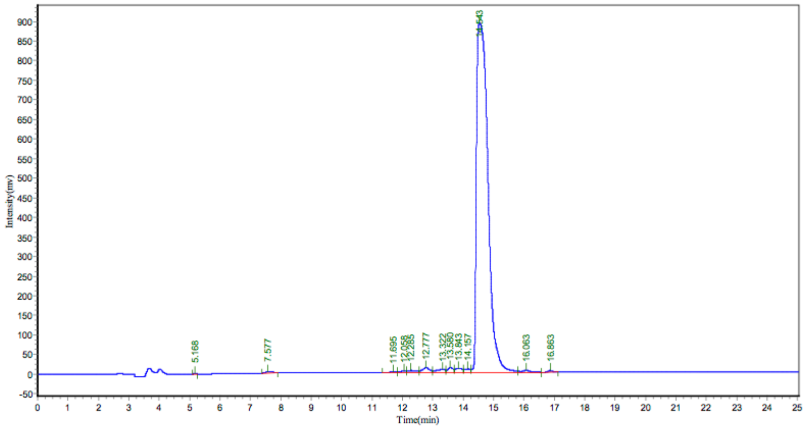

Peak No.	Ret Time	Height	Area	Conc..
1	5.168	989.095	6053.551	0.0248
2	7.577	2928.571	46195.449	0.1894
3	11.695	1712.229	27314.475	0.1120
4	12.058	4593.141	54900.258	0.2251
5	12.285	5001.959	103903.688	0.4260
6	12.777	12315.150	205671.766	0.8432
7	13.322	8183.458	164048.016	0.6726
8	13.580	11724.703	154817.031	0.6347
9	13.843	10584.225	167063.938	0.6849
10	14.157	8972.459	125034.555	0.5126
11	14.543	891739.875	23198542.000	95.1110
12	16.063	5528.797	95069.625	0.3898
13	16.863	4116.423	42399.613	0.1738
Total				100.0000

Supplementary Figure S12. HPLC report for I-P2 (TAMRA-KDPSYSLQVQRQVPV-NH_2). Purity = 95.11%.

Mass Spectrometry Report

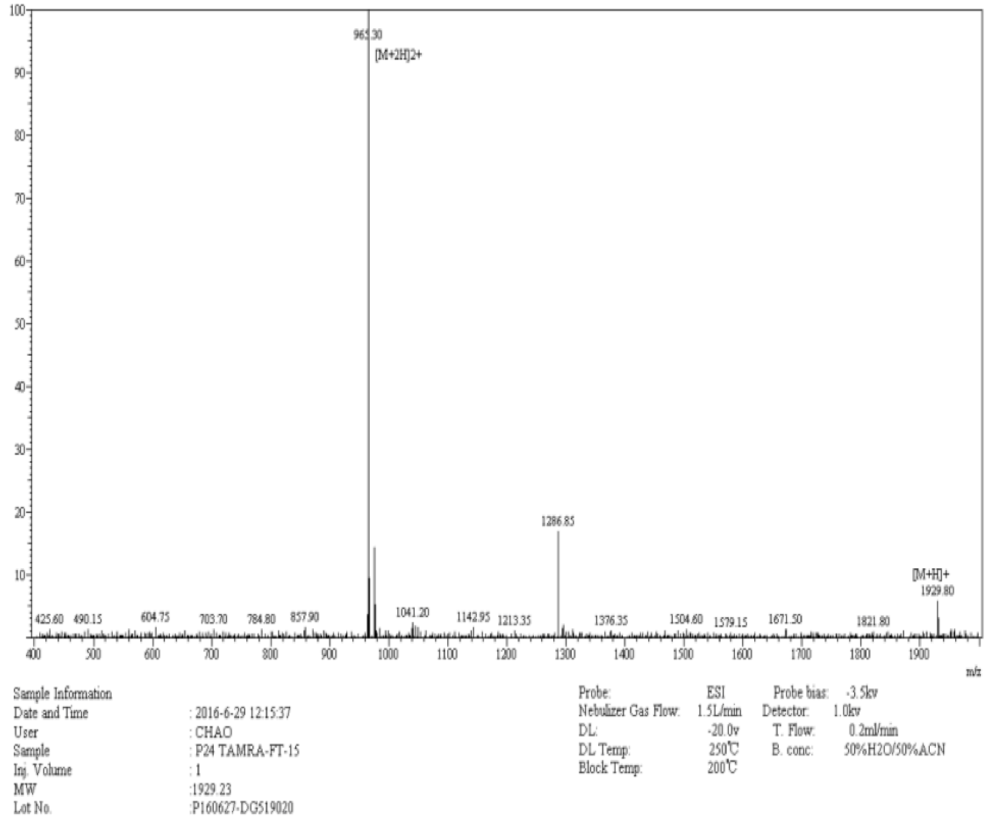

Supplementary Figure S13. Mass spectrometry report for I-P24 (TAMRA-FKGRTSPKTGAPVAT-NH_2). MW = 1929.23 Da.

HPLC Report

Product Name:P24 TAMRA-FT-15
Lot No :P160627-DG519020
Column :4.6*250mm, Kromasil C18-5
Solvent A :0.1%Trifluoroacetic in 100% Acetonirile
Solvent B :0.1%Trifluoroacetic in 100% Water
Gradient :

	A	B
0.01min	21%	79%
25min	46%	54%
25.1min	100%	0%
30min	Stop	

Flow rate :1.0ml/min
Wavelength :220nm
Volume :10ul

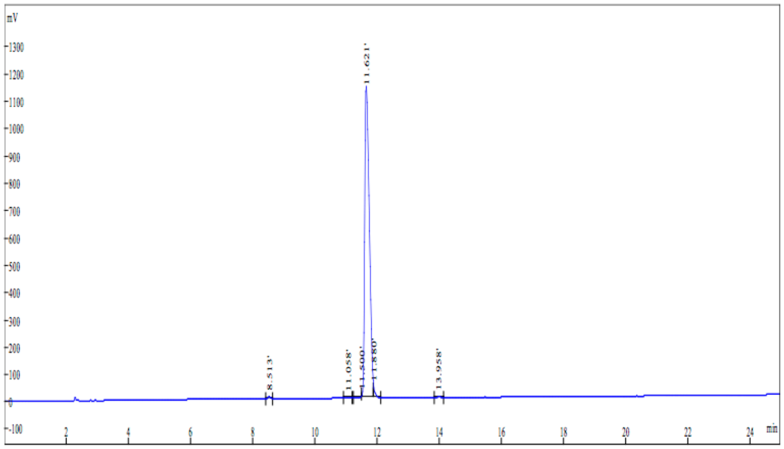

Rank	Time	Conc.	Area	Height
1	8.513	0.3334	40036	6336
2	11.058	0.0888	10665	1665
3	11.500	0.3160	37946	12267
4	11.621	98.3176	11807076	1148719
5	11.880	0.7307	87753	30268
6	13.958	0.2135	25640	4067
Total		100	12009116	1203322

Supplementary Figure S14. HPLC report for I-P24 (TAMRA-FKGRTSPKTGAPVAT-NH_2). Purity = 98.31%.

Mass Spectrometry Report

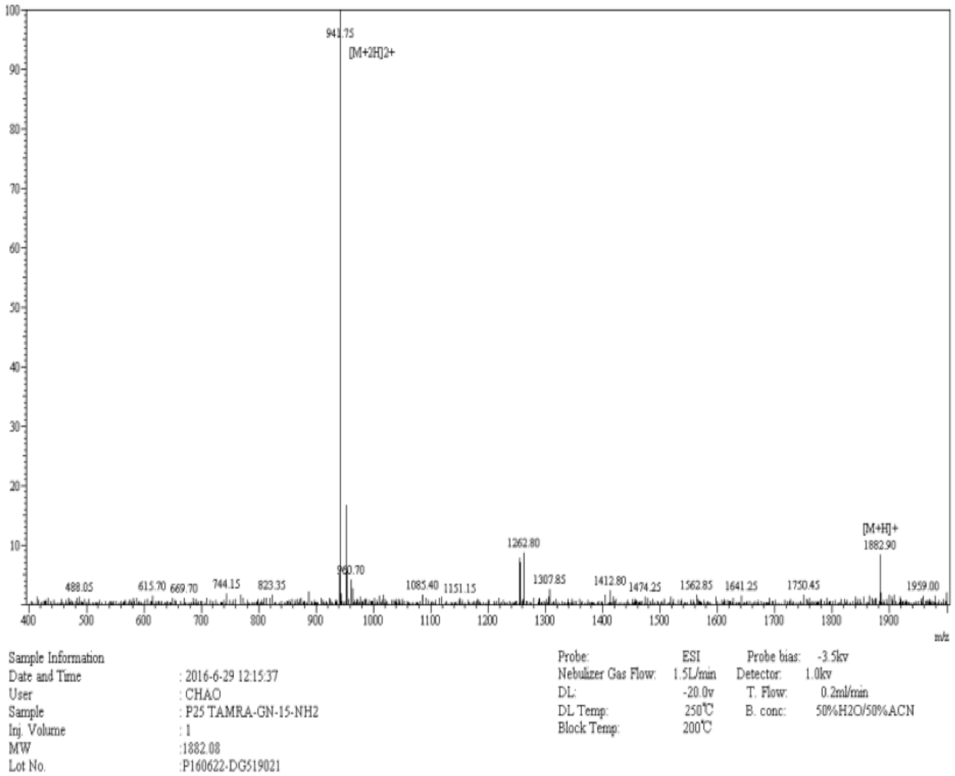

Supplementary Figure S15. Mass spectrometry report for I-P25 (TAMRA-GRTSPKTGAPVATNN-NH_2). MW = 1882.08 Da.

HPLC Report

Product Name:P25 TAMRA-GN-15-NH2
Lot No :P160622-DG519021
Column :4.6*250mm,Kromasil C18-5
Solvent A :0.1%Trifluoroacetic in 100% Acetonirile
Solvent B :0.1%Trifluoroacetic in 100% Water

Gradient :	A	B
0.01min	18%	82%
25min	43%	57%
25.1min	100%	0%
30min	Stop	

Flow rate :1.0ml/min
Wavelength :220nm
Volume :10ul

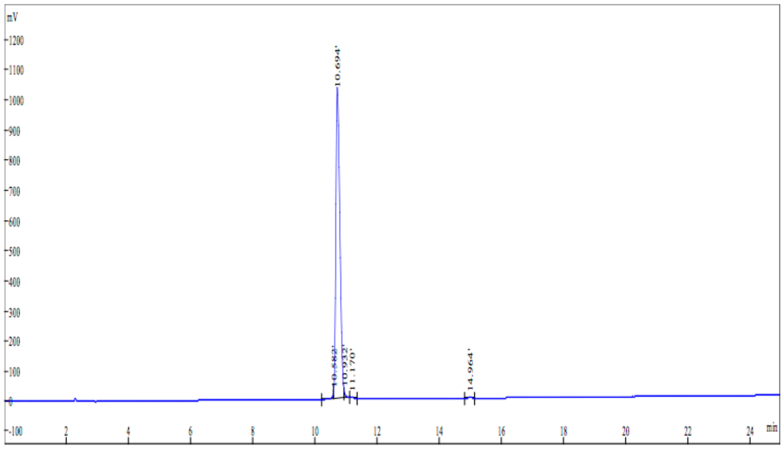

Rank	Time	Conc.	Area	Height
1	10.582	0.7336	69840	28315
2	10.694	97.6547	9296767	1039581
3	10.932	0.8711	82929	18663
4	11.170	0.4634	44114	5994
5	14.964	0.2772	26385	3861
Total		100	9520035	1096414

Supplementary Figure S16. HPLC report for I-P25 (TAMRA-GRTSPKTGAPVATNN-NH_2). Purity = 97.65%.

Mass Spectrometry Report

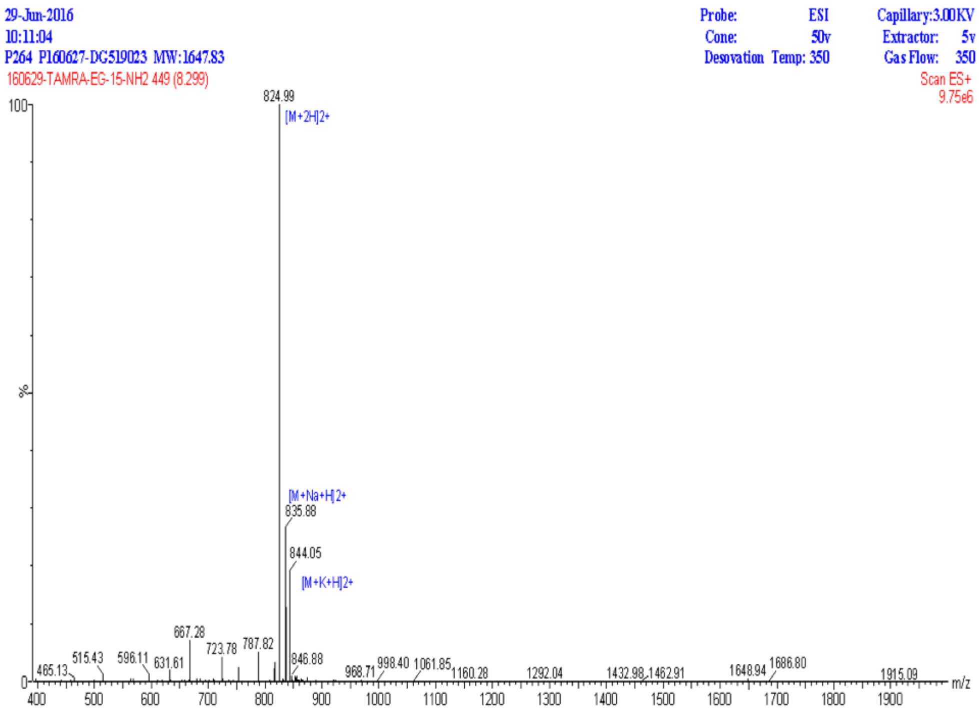

Supplementary Figure S17. Mass spectrometry report for I-P264 (TAMRA-EHGGGLGLGAALGAG-NH_2). MW = 1647.83 Da.

HPLC Report

Structure : P264 TAMRA-EG-15-NH2
Lot No : P160627-DG519023
Column : 4.6×250mm,Venusil MP C18-5
Solvent A : 0.1% trifluoroacetic in 100% acetonitrile
Solvent B : 0.1% trifluoroacetic in 100% water
Gradient :

	A	B
0.01min	28%	72%
25min	53%	47%
25.1min	100%	0%
30min	STOP	

Flow rate : 1.0 mL/min
Wavelength : 220nm
Volume : 5ul

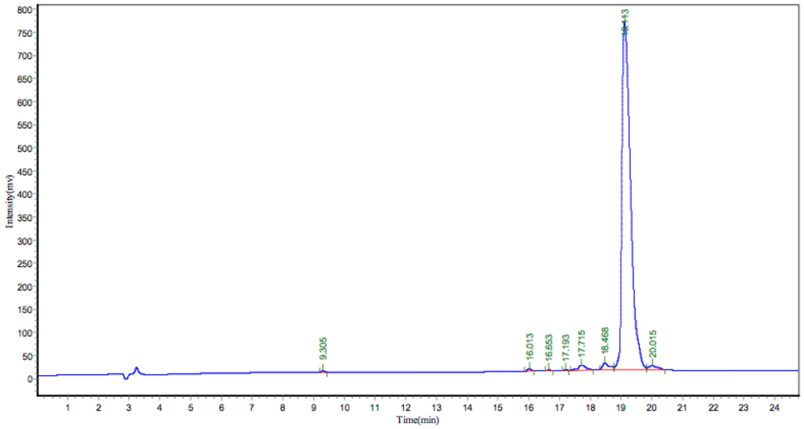

Peak No.	Ret Time	Height	Area	Conc..
1	9.305	2796.874	21745.697	0.1427
2	16.013	4309.023	35781.609	0.2347
3	16.653	1506.251	11383.809	0.0747
4	17.193	1138.502	8025.292	0.0526
5	17.715	9831.731	172064.953	1.1288
6	18.468	14609.321	223201.000	1.4643
7	19.113	753141.438	14592557.000	95.7310
8	20.015	9145.737	178531.891	1.1712
Total				100.0000

Supplementary Figure S18. HPLC report for I-P264 (TAMRA-EHGGGLGLGAALGAG-NH_2). Purity = 95.73%.

Mass Spectrometry Report

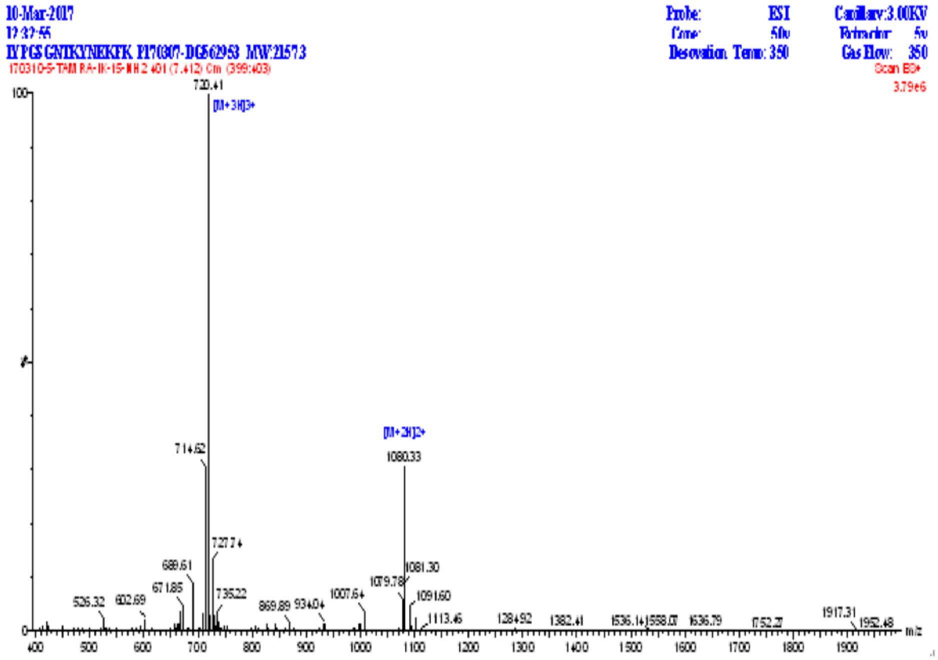

Supplementary Figure S19. Mass spectrometry report for II-P76 (TAMRA-IYPGSGNTKYNEKFK-NH_2). MW = 2157.30 Da.

HPLC Report

Structure : IYPGSGNTKYNEKFK 5-TAMRA-IK-15-NH2
Lot No : P170307-DG562953
Column : 4.6×250mm, Kromasil 100-5-C18
Solvent A : 0.1% trifluoroacetic in 100% acetonitrile
Solvent B : 0.1% trifluoroacetic in 100% water

Gradient :	A	B
0.01min	22%	78%
25min	47%	53%
25.1min	100%	0%
30min	STOP	

Flow rate : 1.0 mL/min
Wavelength : 220nm
Volume : 5ul

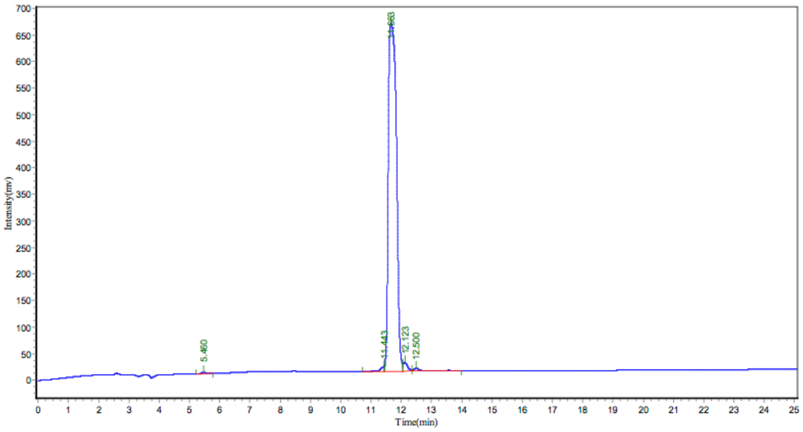

Peak No.	Ret Time	Height	Area	Conc..
1	5.460	2483.306	26077.891	0.2079
2	11.443	9123.130	72267.539	0.5760
3	11.663	655172.438	12183611.000	97.1119
4	12.123	16024.137	174706.813	1.3925
5	12.500	5412.781	89290.938	0.7117
Total				100.0000

Supplementary Figure S20. HPLC report for II-P76 (TAMRA-IYPGSGNTKYNEKFK-NH_2). Purity = 97.11%.

Mass Spectrometry Report

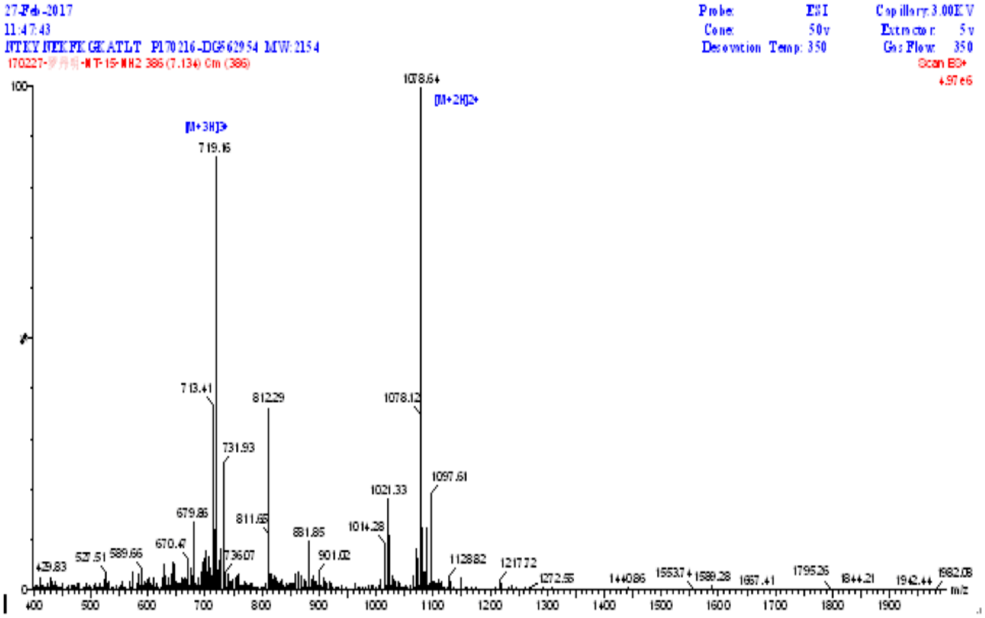

Supplementary Figure S21. Mass spectrometry report for II-P79 (TAMRA-NTKYNEKFKGKATLT-NH_2). MW = 2154.00 Da.

HPLC Report

Product Name	:NTKYNEKFKGKATLT NT-15-NH2
Lot No	:P170216-DG562954
Column	:Gemini-NX 5µ C18 110A, 4.6*250mm
Solvent A	:0.1%Trifluoroacetic in 100% Acetonitrile
Solvent B	:0.1%Trifluoroacetic in 100% Water

Gradient	:	A	B
	0.01min	15%	85%
	25min	40%	60%
	25.01min	100%	0%
	30min	Stop	

Flow rate	:1.0ml/min
Wavelength	:220nm
Volume	:20ul

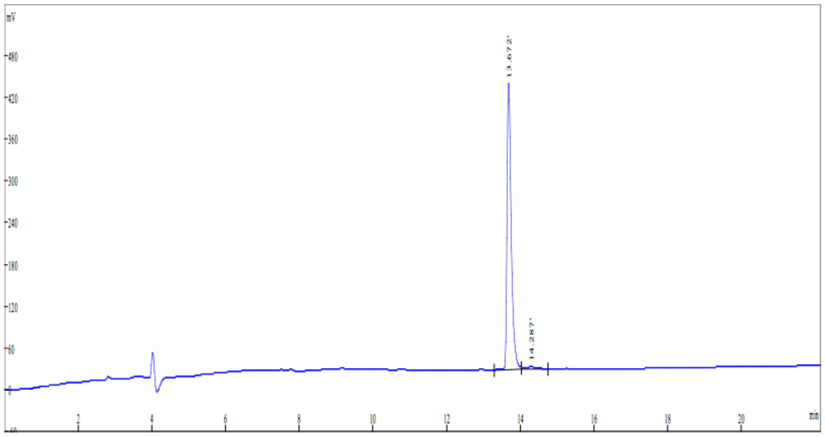

Rank	Time	Conc.	Area	Height
1	13.672	97.5511	2944686	409824
2	14.287	2.4489	73923	3450
Total		100	3018609	413274

Supplementary Figure S22. HPLC report for II-P79 (TAMRA-NTKYNEKFKGKATLT-NH_2). Purity = 97.55%.

Mass Spectrometry Report

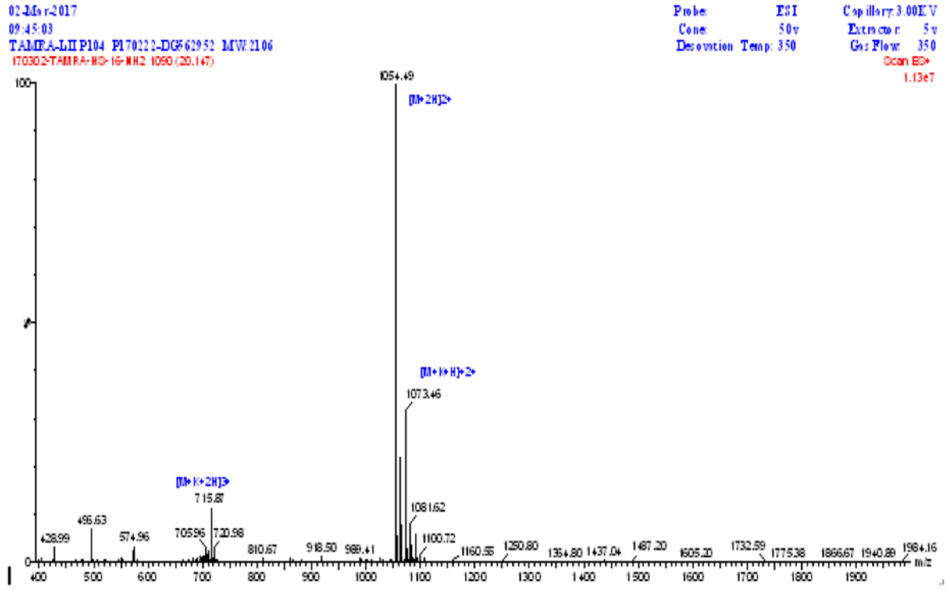

Supplementary Figure S23. Mass spectrometry report for II-P104 (TAMRA-HLSLKNPLRMDLGGGS-NH_2). MW = 2106.00 Da.

HPLC Report

Structure : TAMRA-LII P104 TAMRA-HS-16-NH2
Lot No : P170222-DG562952
Column : 4.6×250mm,Venusil MP C18-5
Solvent A : 0.1% trifluoroacetic in 100% acetonitrile
Solvent B : 0.1% trifluoroacetic in 100% water
Gradient :

	A	B
0.01min	25%	75%
25min	50%	50%
25.1min	100%	0%
30min	STOP	

Flow rate : 1.0 mL/min
Wavelength : 220nm
Volume : 5ul

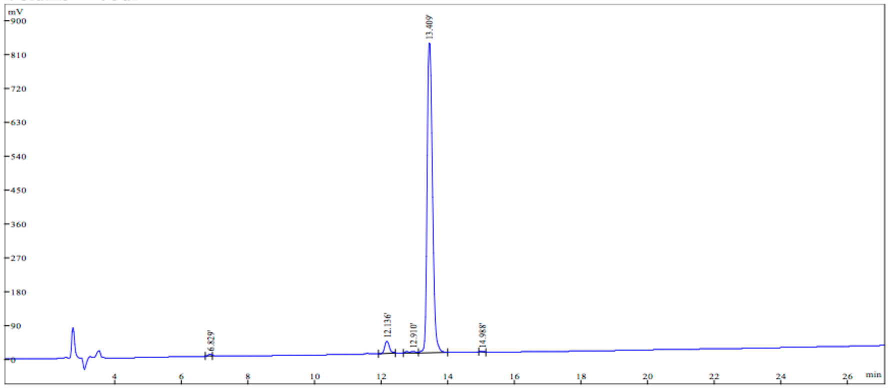

Rank	Time	Conc.	Area	Height
1	6. 829	0. 3995	33099	4897
2	12. 136	3. 36	278327	31350
3	12. 910	0. 6398	53002	3708
4	13. 409	95. 47	7908055	823409
5	14. 988	0. 1352	11200	1526
Total		100	8283683	864890

Supplementary Figure S24. HPLC report for II-P104 (TAMRA-HLSLKNPLRMDLGGGS-NH_2). MW = 95.47%.

MS REPORT

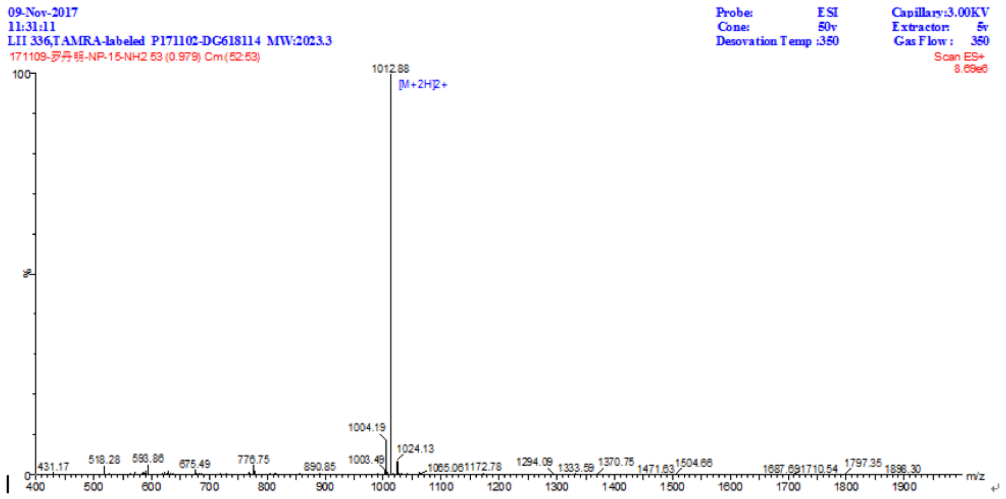

Supplementary Figure S25. Mass spectrometry report for II-P336 (TAMRA-NRTVLRNLGNGTSLP-NH_2). MW = 2023.30 Da.

HPLC REPORT

Product Name	:LII 336, TAMRA-labeled NP-15-NH2
Lot No	:P171102-DG618114
Column	:Gemini-NX 5µ C18 110A, 4.6*250mm
Solvent A	:0.1%Trifluoroacetic in 100% Acetonitrile
Solvent B	:0.1%Trifluoroacetic in 100% Water
Gradient	: A B
	0.01min 15% 85%
	25min 40% 60%
	25.01min 100% 0%
	30min Stop
Flow rate	:1.0ml/min
Wavelength	:220nm
Volume	:20ul

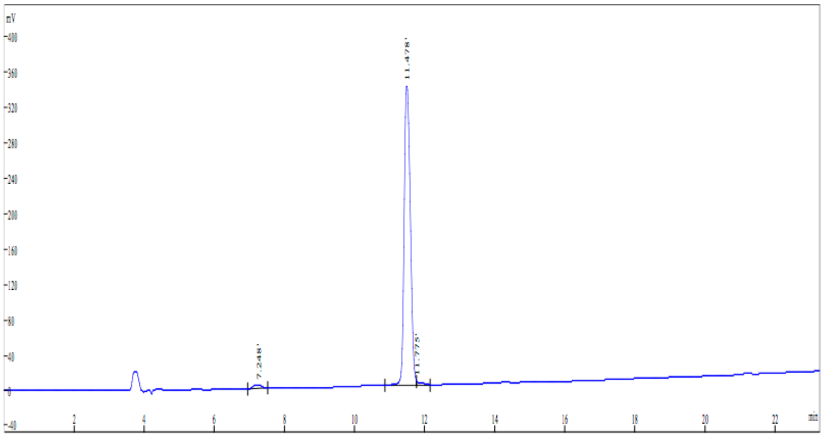

Rank	Time	Conc.	Area	Height
1	7.248	1.6307	64095	3806
2	11.478	97.5453	3833977	337016
3	11.775	0.8240	32389	3531
Total		100	3930461	344353

Supplementary Figure S26. HPLC report for II-P336 (TAMRA-NRTVLRNLGNGTSLP-NH_2). Purity = 97.54%.

Mass Spectrometry Report

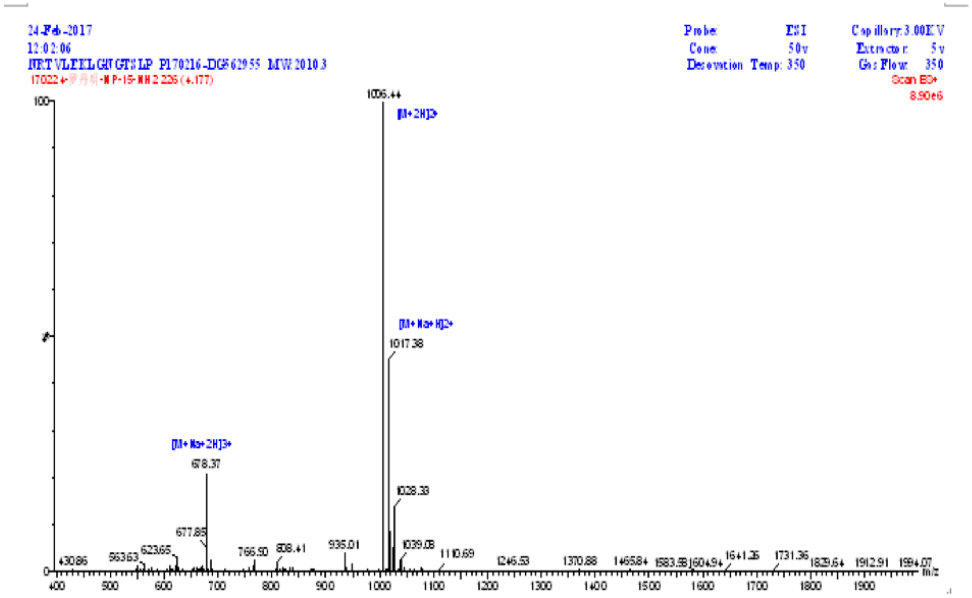

Supplementary Figure S27. Mass spectrometry report for II-P341 (TAMRA-NRTVLEKLGNGTSLP-NH_2). MW = 2010.30 Da.

HPLC Report

Product Name	:NRTVLEKLGNGTSLP NP-15-NH2
Lot No	:P170216-DG562955
Column	:Gemini-NX 5μ C18 110A, 4.6*250mm
Solvent A	:0.1%Trifluoroacetic in 100% Acetonitrile
Solvent B	:0.1%Trifluoroacetic in 100% Water

Gradient	:	A	B
	0.01min	20%	80%
	25min	45%	55%
	25.01min	100%	0%
	30min	Stop	

Flow rate	:1.0ml/min
Wavelength	:220nm
Volume	:20ul

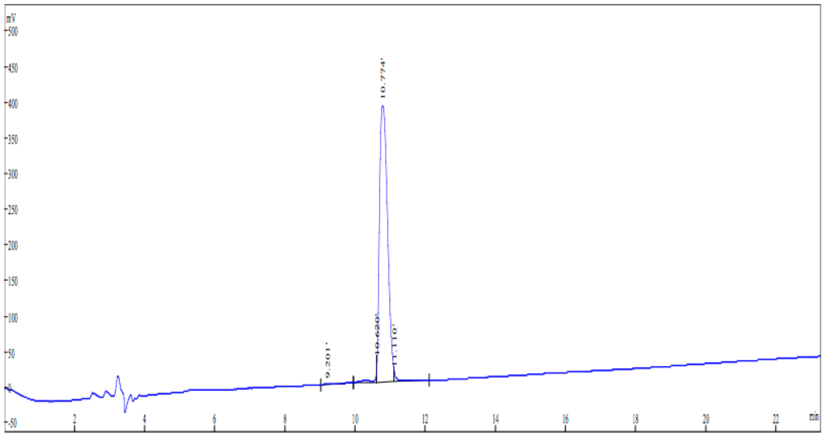

Rank	Time	Conc.	Area	Height
1	9.201	0.4679	28543	1436
2	10.620	1.9139	116756	29088
3	10.774	96.3465	5877574	387341
4	11.110	1.2717	77581	11873
Total		100	6100454	429738

Supplementary Figure S28. HPLC report for II-P341 (TAMRA-NRTVLEKLGNGTSLP-NH_2). Purity = 96.34%.

S5. Rheology for colominic acid viscosity assessment

To understand the effect of colominic acid (CA) viscosity on fluorescence anisotropy experiments, rheology was used to determine the dependence of CA concentration on solution viscosity (Fig. S29). Viscosity of CA solutions from 0.15–1500 μM CA in PBS, pH 7.4 (10 mM phosphate, 100 mM NaCl) was measured at 22 °C using an Anton Paar MCR 301 rheometer with shear rates from 1–2000 s^{-1}. Since viscosity did not vary with increasing shear rate, CA solutions at relevant concentrations were assumed to be Newtonian, and hence viscosity at different concentrations was directly assessed from *y*-intercepts of viscosity over shear rate profiles.

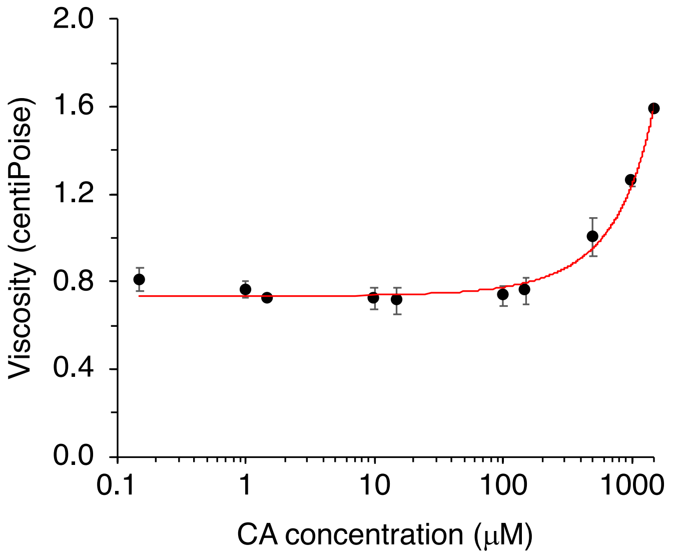

Supplementary Figure S29. Change in CA solution viscosity with concentration. Error bars represent standard deviation of the mean from two independent experiments (measurements repeated three times in one of the two experiments). The trendline shown is from a transformed exponential function to demonstrate the exponential trend in viscosity and does not represent a non-linear fit (R^2 of transformed function = 0.97).

S6. References for supplementary information

1. Ambrosi, M., Cameron, N. R. & Davis, B. G. Lectins: tools for the molecular understanding of the glycocode. *Org. Biomol. Chem.* **3**, 1593–1608 (2005).
2. Gabius, H. J., André, S., Jiménez-Barbero, J., Romero, A. & Solís, D. From lectin structure to functional glycomics: principles of the sugar code. *Trends Biochem. Sci.* **36**, 298–313 (2011).
3. Weis, W. I. & Drickamer, K. Structural basis of lectin–carbohydrate recognition. *Annu. Rev. Biochem.* **65**, 441–473 (1996).
4. Stoolman, L. M. & Rosen, S. D. Possible role for cell-surface carbohydrate-binding molecules in lymphocyte recirculation. *J. Cell Biol.* **96**, 722–729 (1983).
5. Lipfert, J., Doniach, S., Das, R. & Herschlag, D. Understanding nucleic acid–ion interactions. *Annu. Rev. Biochem.* **83**, 813–841 (2014).
6. Häyrinen, J. *et al*. High affinity binding of long-chain polysialic acid to antibody, and modulation by divalent cations and polyamines. *Mol. Immunol.* **39**, 399–411 (2002).
7. Kanato, Y., Kitajima, K. & Sato, C. Direct binding of polysialic acid to a brain-derived neurotrophic factor depends on the degree of polymerization. *Glycobiology* **18**, 1044–1053 (2008).
8. Nagae, M. *et al*. Crystal structure of anti-polysialic acid antibody single chain Fv fragment complexed with octasialic acid: insight into the binding preference for polysialic acid. *J. Biol. Chem.* **288**, 33784–33796 (2013).
9. He, W. *et al*. Production of chondroitin in metabolically engineered *E. coli*. *Metab. Eng.* **27**, 92–100 (2015).
10. González-Clemente, C., Luengo, J. M., Rodríguez-Aparicio, L. B., Ferrero, M. A. & Reglero, A. High production of polysialic acid [Neu5Acα(2-8)-Neu5Acα(2- 9)]$_n$ by *Escherichia coli* K92 grown in a chemically defined medium: regulation by temperature. *Biol. Chem. Hoppe-Seyler* **371**,1101–1106 (1990).
11. Fu, L. *et al*. Structural characterization of pharmaceutical heparins prepared from different animal tissues. *J. Pharm. Sci*. **102**, 1447–1457 (2013).
12. Shastry, D. G. & Karande, P. Microarrays for the screening and identification of carbohydrate-binding peptides. *Analyst* **144**, 7378–7389 (2019).

13. Röckendorf, N., Bade, S., Hirst, T. R., Gorris, H. H. & Frey, A. Synthesis of a fluorescent ganglioside G_{M1} derivative and screening of a synthetic peptide library for G_{M1} binding sequence motifs. *Bioconj. Chem.* **18**, 573–578 (2007).
14. Rode, B. *et al*. Large-scale production and homogenous purification of long chain polysialic acids from *E. coli* K1. *J. Biotech.* **135**, 202–209 (2008).
15. Patane, J., Trapani, V., Villavert, J. & McReynolds, K. D. Preparative production of colominic acid oligomers via a facile microwave hydrolysis. *Carbohydr. Res.* **344**, 820–824 (2009).
16. Siebert, H.-C. *et al*. Interaction studies of sialic acids with model receptors contribute to nanomedical therapies. *J. Neurol. Disord.* **3**, 1000212, https://doi.org/10.4172/2329-6895.1000212 (2015).
